# Integrating Network Pharmacology and Experimental Validation Deciphers the Mechanism of Guizhi Fuling Wan against Adenomyosis

**DOI:** 10.1155/2021/6034147

**Published:** 2021-10-26

**Authors:** Haoxian Wang, Jihong Zhang, Qinqin Zhu, Xianyun Fu, Chenjie Li

**Affiliations:** ^1^The Second People's Hospital of Yichang, YChina Three Gorges University, Yichang 443000, China; ^2^Medical College, China Three Gorges University, Yichang 443002, China

## Abstract

**Aim:**

This study aimed to predict the key targets and endocrine mechanisms of Guizhi Fuling Wan (GZFLW) in treating adenomyosis (AM) through network pharmacology, molecular docking, and animal experiment verification.

**Methods:**

The related ingredients and targets of GZFLW in treating AM were screened out using TCMSP, BATMAN-TCM, SwissTargetPrediction, and PubChem Database. Then, the protein-protein interaction (PPI) analysis and the network of compound-hub targets were constructed. At the same time, the key targets were uploaded to the Metascape Database for KEGG pathway enrichment analysis. After that, the molecular docking technology of the main active components and hub targets was performed. Furthermore, animal experiments were used to verify the results of network pharmacology analysis.

**Results:**

A total of 55 active ingredients of GZFLW and 44 overlapping targets of GZFLW in treating AM were obtained. After screening, 25 hub targets were collected, including ESR1, EGF, and EGFR. Then, the KEGG pathway enrichment analysis results indicated that the endocrine therapeutic mechanism of GZFLW against AM is mainly associated with the estrogen signaling pathway, endocrine resistance, and an EGFR tyrosine kinase signaling pathway. Then, molecular docking showed that the significant compounds of GZFLW had a strong binding ability with ER*α* and EGFR. More importantly, the animal experiments confirmed that the GZFLW could downregulate the abnormal infiltration of the endometrial epithelium into the myometrium and had no interference with the normal sexual cycle. This effect may be directly related to intervening the local estrogen signaling pathway of the endometrial myometrial interface (EMI). It may also be associated with the myometrium cells' estrogen resistance via GPER/EGFR signaling pathway.

**Conclusion:**

The endocrine mechanism of GZFLW in treating AM was explored based on network pharmacology, molecular docking, and animal experiments, which provided a theoretical basis for the clinical application of GZFLW.

## 1. Introduction

In 2020, 1.03% of the women were suffering from adenomyosis (AM), and approximately 90% of them were accompanied by menorrhagia, progressive dysmenorrhea, pelvic pain, dysmenorrhea, and infertility [1]. AM is characterized by the progressive invasion of endometrial cells into the myometrium, attributed to endocrine disorders, chronic hypoxia, and inflammation in the endometrial myometrial interface (EMI) [2, 3]. Current therapeutic approaches, including operative treatment, nonsteroidal anti-inflammatory drugs, and endocrine therapy, have been used to suppress inflammation, relieve pain, and improve fertility. However, several limitations including poor patients' response, high prices, and long-term side effects [4, 5]. Therefore, it is of great clinical significance to develop alternative therapeutic strategies to improve the outcome of AM patients.

Traditional Chinese medicine (TCM), which has been widely used in clinical practice in China for thousands of years, has made considerable contributions to the relief of AM [6]. Guizhi Fuling Wan (GZFLW) is an effective Chinese medicinal formula for AM which is used in TCM [7, 8]. This medication consists of the following five herbs: Cinnamon Twig (CT), *Poria cocos* (PC), Cortex Moutan (CM), Radix Paeoniae Rubra (RPR), and Peach Kernel (PK). The complex composition of GZFLW makes it not easy to conduct mechanistic studies on it. Thus, the “multicomponent and multitarget” therapeutic mechanism of GZFLW in treating AM is still obscure, hindering its clinical use. Hence, for a comprehensive understanding of the mechanism of action of GZFLW, a novel analysis method is needed [9].

In this study, a network pharmacology strategy was adopted for investigating the mechanism of GZFLW against AM. We screened representative compounds in five herbs of GZFLW. Their potential targets in treating AM were identified. Furthermore, molecular docking technology was used to verify the binding ability of active ingredients to several potential targets. In addition, animal experiments have further confirmed some of the hypotheses. The results revealed that GZFLW might modulate the estrogen signaling pathway, endocrine resistance, and EGFR tyrosine kinase signaling pathway to exert an effect. These results may provide novel sights into the anti-AM effect of GZFLW.

## 2. Materials and Methods

### 2.1. Chemical Ingredients Database Building

The active ingredients of the five herbs contained in GZFLW were obtained using the Traditional Chinese Medicine Systems Pharmacology Database (TCMSP, http://lsp.nwu.edu.cn/tcmsp.php) and the Bioinformatics Analysis Tool for Molecular Mechanism of Traditional Chinese Medicine (BATMAN-TCM, http://bionet.ncpsb.org/batman-tcm/index.php), which are both bioinformatics analysis tools for the main components of TCM.

### 2.2. Active Ingredients Screening

The oral bioavailability (OB) and drug-likeness (DL) were used as screening indexes. OB is the percentage of oral drugs absorbed into the systemic circulation, which is a frequently used pharmacokinetic parameter. DL refers to the property of measuring the absorption, distribution, metabolism, and excretion (ADME) of drug molecules, which could help optimize pharmacokinetic and pharmaceutical properties. The OB and DL indices of all the related ingredients are presented in the TCMSP. We set the indexes of OB ≥ 30% and DL ≥ 0.18 as the screening criteria of active ingredients. Briefly, components that satisfied the screening threshold of OB and DL were regarded as the candidate compounds.

### 2.3. GZFLW Targets Prediction

The targets of active ingredients were searched in TCMSP, BATMAN-TCM, SwissTargetPrediction Database (http://www.swisstargetprediction.ch/index.php), and PubChem Database (https://pubchem.ncbi.nlm.nih.gov/), with the species limited as ‘‘*Homo sapiens*.”

### 2.4. Adenomyosis Therapeutic Targets Prediction

Targets associated with AM were collected by GeneCards Database (https://www.genecards.org/) and Online Mendelian Inheritance in Man Database (OMIM, https://www.omim.org). We searched these databases with the keyword “adenomyosis” to obtain related targets. After deletion of duplications, candidate targets against AM were identified by matching the targets of the active ingredients of GZFLW and the genes related to AM. These targets were then uploaded to STRING Database (https://string-db.rog/) to obtain the protein-protein interaction (PPI) information between two proteins or multiple proteins with the confidence ≥0.7.

### 2.5. Network Topological Feature Set Definitions

For each node in the interaction network, we selected two parameters to evaluate its topological features: “Degree” reflects the number of other nodes interacting with this node; the percentage of all shortest paths measures “Betweenness Centrality” (BC). Nodes greater than or equal to the median are screened as hub nodes for GZFLW against AM.

### 2.6. KEGG Enrichment Analysis

We used the Metascape Database (http://metascape.org/) to obtain the potential pathways of GZFLW in treating AM. *P* < 0.05 was regarded as the cutoff value.

### 2.7. Molecular Docking of Hub Targets and Active Ingredients

Molecular docking is a computational tool to predict the binding ability and connection type of proteins and ligands. It can calculate and predict the conformation and directions of ligands at active protein sites. AutoDock 1.5.6 (http://autodock.scripps.edu/) was a molecular docking software for network pharmacology based prediction and analysis, which could be used to dock the hub targets and active ingredients. In this process, structures files in the formats of 3D of hub targets we chose were obtained from RCSB Protein Data Bank (PDB, http://www.rcsb.org/): EGFR (PDB ID: 2GS2) and ER*α* (PDB ID: 6CHW); structures files in the formats of 3D of active compounds were provided from ZINC Database (http://zinc.docking.org/) and PubChem Database (https://pubchem.ncbi.nlm.nih.gov/). During the docking process, the PDBQT files of these structures were used in AutoDock software, as these files contained the information of potential ligands and receptors required for the process. After that, the Lamarckian genetic algorithm was used to obtain docking results and the binding energy of ligands and receptors. At last, the results were output in .dlg files and uploaded to PyMOL software for establishing the 3D results binding models. Binding energy was used as a docking score to evaluate the protein-ligand binding potential of active ingredients to hub targets. Among them, those results with value ≤ −5 were selected and considered to have moderate binding potential and tight combination.

### 2.8. HPLC and Mid-Infrared Spectroscopy Analysis

The HPLC finger printing of CT, RPR, PK, and CM in GZFLW was constructed using Series LC-2010A Liquid Chromatograph (Shimadzu, Japan). Octadecyl silane bonded silica was used as the stationary phase (column: hypersil ODS C18 4.6 mm × 250 mm, 5 *μ*m). The mobile phase was ammonium acetate (A) and water (B). The flow rate was kept constant at 1 mL/min. The system operated at 30°C and the injection volume was 10 *μ*L (CT, RPR, and PK) and 5 *μ*L (CM). The detection wavelength was, respectively, kept at 285 nm (CT), 230 nm (RPR), 274 nm (CM), and 210 nm (PK). The reference substances (cinnamic acid, cinnamaldehyde, gallic acid, catechin, paeonol, paeoniflorin, and amygdalin) were prepared by Chengdu Ruifensi Biotechnology Co., Ltd. (Chengdu, China).

The active ingredients of PC were analyzed by mid-infrared spectroscopy. The ingredient was weighed, adding about 100 mg KBr powder which was dried at 120°C for 4 h. Then, grind and mix well in the mortar, transfer to the mold, and press the samples into transparent slices at a pressure of about 30 MPa for 1∼2 min. The sample slice thickness was about 1∼2 mm. The Fourier transform infrared spectrometer (deuterated triglycine sulfate detector) was used with a spectral range of 4000∼400 cm^−1^. Each sample was repeated twice.

### 2.9. In Vivo Experiment

Virgin female ICR mice (26–29 g, 7 weeks old) were provided by the Experimental Animals Center of China Three Gorges University. All the animals were housed at a temperature of 22 ± 2°C, humidity of 55–65%, and light controlled (12 h light/12 h dark cycle) vivarium, with food and water ad libitum. Animal welfare and experimental procedures were carried out in accordance with the Guide for the Care and Use of Laboratory Animals and were approved by the China Three Gorges University Medical Animal Ethics Commission (Approval No. 20190801).

Twenty-five mice were implanted with a single pituitary gland each under the capsule of the right uterus at 7 weeks of age and then were divided randomly into 2 groups: model group (*n* = 14) and GZFLW group (*n* = 11). After intraperitoneally anesthetizing with emulsion (Xian Nippon Pharmaceutical Co., Ltd., Xian, China, No. H19990282, 100 mg/kg), pituitary obtained from age-matched male mice was grafted into the right uterine lumen in female mice. Then, the gentamycin solution (0.25 ml, 20000 units/20 g) was dropped intraperitoneally to prevent infection. Mice receiving no pituitary grafts served as controls (*n* = 8). One day after the pituitary grafting, 11 mice were intragastriced with decocting-free granules of GZFLW (4.25 mg/g of body weight, consisting of CT, PC, CM, RPR, and PK at the ratio of 1 : 1:1 : 1:1, Beijing Kangrentang Pharmaceutical Co., Ltd., Beijing, China, Nos. 19026231, 19027291, 19019661, 19004891, and 19006412) and dissolved in aqueous solution every day for 8 weeks (GZFLW group). The remaining 22 mice (model group 14, control group 8) were intragastriced with the same dosage of saline as a control vehicle. All mice were killed at 15 weeks of age by decapitation, after the estrous cycle had been checked by vaginal smear.

Haematoxylin and eosin (HE) staining was used to observe the depth of invasion. Immunofluorescence was adopted to identify the expressions of ER*α*, GPER, and EGFR in the glandular, stromal, and muscular parts in the endometrial myometrial interface (EMI) (magnification, 200×, every microscope field taking three different parts for analysis).

### 2.10. Data and Statistical Analysis

Data were analyzed using SPSS 20 software and presented as mean ± standard deviation (SD). Statistical differences between groups were evaluated by one-way analysis of variance (ANOVA). The Chi-square test was applied to analyze the categorical variables. Pearson analysis was used for correlation. *P* < 0.05 was regarded as a statistically significant difference.

## 3. Results and Discussion

### 3.1. Active Ingredients and Targets of GZFLW

By retrieving from the databases, there were 498 related ingredients of GZFLW in total: 222 ingredients in CT, 33 ingredients in PC, 56 ingredients in CM, 121 ingredients in RPR, and 66 ingredients in PK. After eliminating the redundancy and screening with OB and DL, 55 active components and 364 corresponding targets of GZFLW were obtained. It is worth noting that cinnamaldehyde (OB = 31.99%, DL = 0.02), cinnamic acid (OB = 19.68%, DL = 0.03), benzoic acid (OB = 31.55%, DL = 0.02), paeonol (OB = 28.79%, DL = 0.04), gallic acid (OB = 31.69, DL = 0.04), and amygdalin (OB = 4.42%, DL = 0,61) have a relatively low OB or DL value, but they were included in this study because they were the vital pharmacological compounds identified, which were confirmed to the previous study (Table 1) (Table S1).

### 3.2. Target Analysis

**Figure 1:**
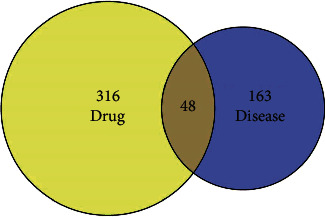
The Venn diagram of the targets in both endometriosis targets and GZFLW targets.

Via the keyword of “adenomyosis,” the therapeutic targets of AM were obtained from GeneCards and OMIM Database with a total of 211 (Table S2). A total of 48 overlapping targets were then obtained as the related targets of GZFLW in treating AM (Figure 1).

**Figure 2:**
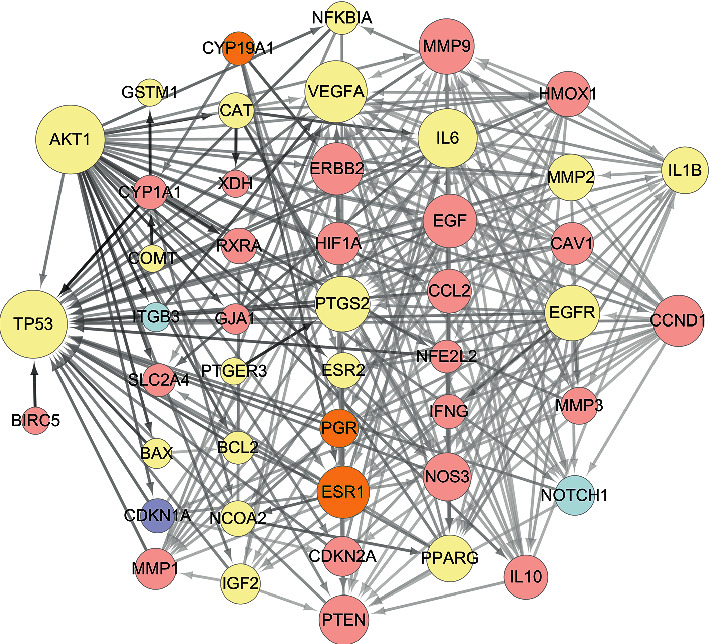
The network of 48 nodes. The different color represents different nodes from each ingredient: The blue nodes represent the targets from CT, the pink nodes represent the targets from CM, the purple nodes represent the targets from RPR, the orange nodes represent the targets from PC, and the yellow nodes represent the targets that are targeted by more than one ingredient. The node size is proportional to the target degree in the network. The edge color changes from light to dark reflect that the betweenness value changes from low to high in the network.

**Figure 3:**
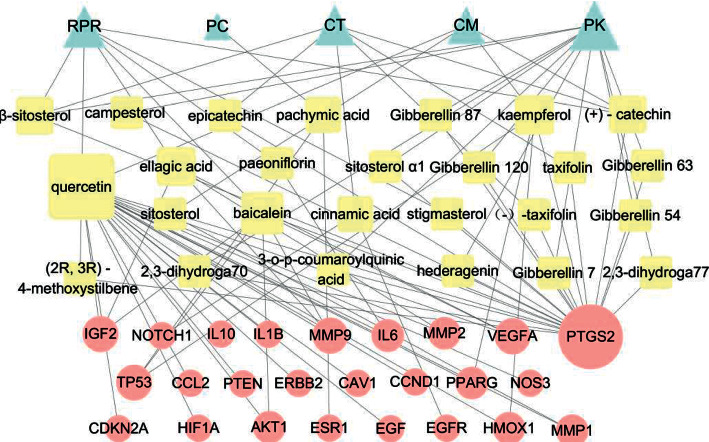
Herbs-Compounds-Hub Targets Network. The triangle nodes represent herbs, the square nodes represent active ingredients, and the circle nodes represent key targets. RPR represents Radix Paeoniae Rubra, PC represents *Poria cocos*, CT represents Cinnamon Twig, CM represents Cortex Moutan, and PK represents Peach Kenel. The node size is proportional to the target degree in the network.

From the STRING Database of “multiple proteins” interaction of targets, the data of the protein-protein interaction (PPI) network of those 48 targets were subsequently obtained (Table S3). With confidence ≥0.7, we gained 268 edges of 48 nodes. With betweenness ≥14 and degree ≥0.01, the topological analysis of targets mentioned above was performed. The results were 25 hub nodes with 183 edges, in which 25 nodes represented 25 central targets, including ESR1, EGF, and EGFR. When the 25 significant nodes and the other 23 nodes were distributed with “degree” and “betweenness,” the network of 48 nodes was constructed as in Figure 2. The comprehensive network of “Herbs-Compounds-Hub Targets” had 25 nodes related to 26 ingredients, as determined by the Cytoscape 3.7.1 software (Figure 3).

### 3.3. KEGG Pathway Enrichment Analysis

**Figure 4:**
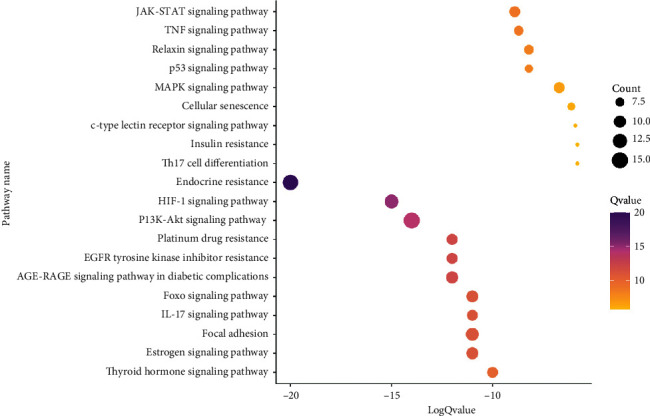
Information of 20 pathways about KEGG enrichment.

In the Metascape Database, we performed the KEGG pathway enrichment analysis on 48 targets and obtained 114 pathways (Table S4). We ranked these pathways according to the *P*-value of each enriched pathway in ascending order, and the top 20 pathways were selected, which mainly related to endocrine resistance, EGFR tyrosine kinase inhibitor resistance, and estrogen signaling pathway (Figure 4).

### 3.4. Molecular Docking

**Figure 5:**
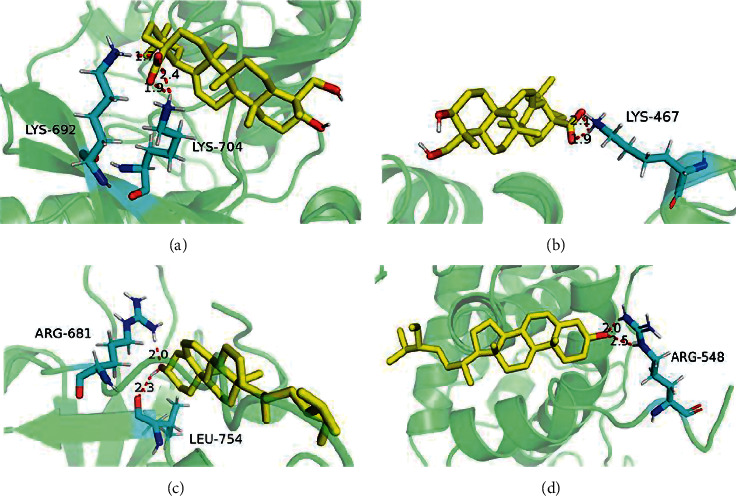
The network of molecular docking results. (a) EGFR-hederagenin. (b) ER*α*-hederagenin. (c) EGFR-*β*-sitosterol. (d) ER*α*-*β*-sitosterol. The yellow sticks represent EGFR and ER*α*. The blue sticks represent active compounds.

To further explore the binding ability of active components and hub targets in GZFLW, molecular docking technology was used. Due to the fact that ER*α* and EGFR were the core targets of both estrogen pathway and endocrine resistance pathway, the two were selected to bind to active ingredients. 71.43% of the compounds (10/17) had binding energies lower than −5, which verified that those active components may be well binding with ER*α* and EGFR (Table 2). The 3D binding mode of 2 major active ingredients in the active site of ER*α* and EGFR, respectively, is represented in Figure 5.

### 3.5. HPLC Analysis of CT, CM, RPR, and PK

**Figure 6:**
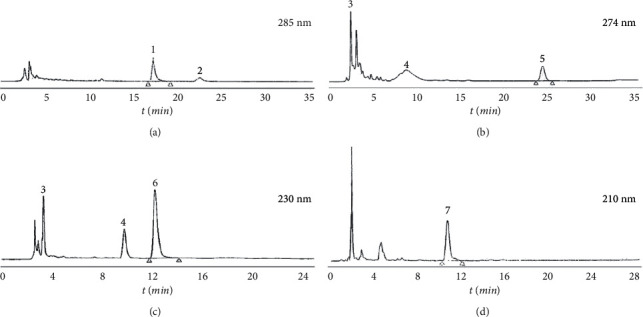
HPLC fingerprint of CT, CM, RPR, and PK. (a) Cinnamon Twig (CT). (b) Cortex Moutan (CM). (c) Radix Paeoniae Rubra (RPR). (d) Peach Kernel (PK). 1: cinnamic acid; 2: cinnamaldehyde; 3: gallic acid; 4: (+)-catechin; 5: paeonol; 6: paeoniflorin; 7: amygdalin.

There were six main peaks in the fingerprint of CT at 285 nm, and two active components were determined after comparison with standards; the ten main peaks in the fingerprint of CM at 274 nm were obtained, and three vital components were confirmed; there were five main peaks in the fingerprint of RPR at 230 nm, and, among these, three active ingredients were determined; the results in the fingerprint of PK at 210 nm were four main peaks, defining one significant ingredient (Figure 6). All of the main peaks had good separation.

### 3.6. Mid-Infrared Spectroscopy

**Figure 7:**
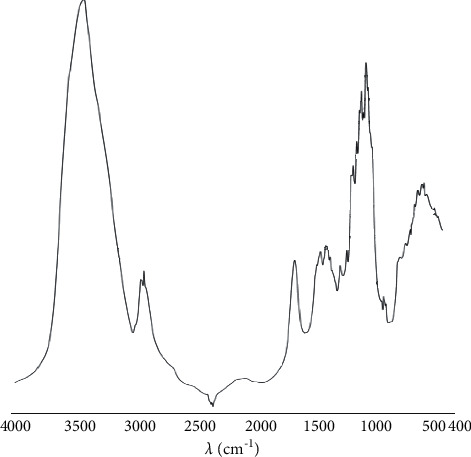
IR spectra of PC.

Around 3400 cm^−1^, these frequencies were associated with O–H stretching. Around 2800 cm^−1^, the peaks of C-H antisymmetric stretching of methylene group were observed. Frequencies around 1600 cm^−1^ and 1450 cm^−1^ were associated with O-H and C-H bends. Below 1300 cm^−1^, the frequencies were associated with C-O stretching, with frequencies between 1200 and 1000 cm^−1^ showing the presence of polysaccharide. In addition, frequencies below 950 cm^−1^ were related to sugar ring skeleton stretching (Figure 7).

### 3.7. Effect on Infiltration Depth in Mice

**Figure 8:**
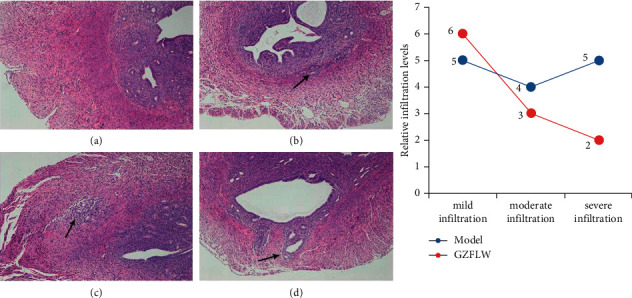
HE staining of the transverse section of the uterus. (a) Normal transverse section of the uterus. (b) Mild infiltration. (c) Moderate infiltration. (d) Severe infiltration. Arrows indicate infiltration of endometrial glands and stroma into the inner myometrium (b), outer myometrium (c), and serosa (d).

AM model was successfully established after pituitary grafts, and HE staining indicated that endometrial stroma and glandular epithelial cells infiltrated into the myometrium. According to the infiltration depth, the models can be divided into mild, moderate, and severe infiltration (Figure 8). The results showed that the proportion of moderate and severe infiltration decreased after GZFLW treatment.

### 3.8. Effect on the Sexual Cycle in Mice

**Figure 9:**
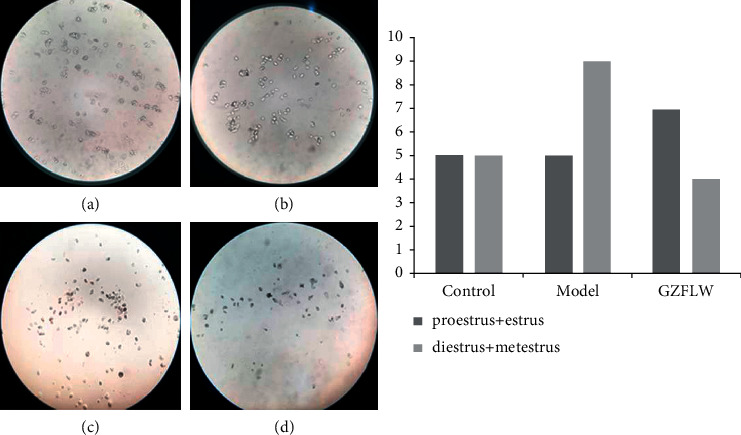
Observation of vaginal smears and sexual cycle statistics of mice. (a) Proestrus. (b) Estrus. (c) Metestrus. (d) Diestrus.

The estrous cycle of mice can be divided into four stages: proestrus (mainly nucleated epithelial cells), estrus (mainly nonnucleated keratinocytes), metestrus (mainly a small number of keratinocytes and more leukocytes), and diestrus (mainly a large number of leukocytes). We divided the estrous cycles into two phases, combining metestrus with diestrus because of the leucocytic predominance [10]. There is no significant difference in vaginal smears among the three groups by Chi-square test, which confirmed that GZFLW treatment had no critical interference in the sexual cycle of mice (Figure 9).

### 3.9. Immunofluorescence Assay

**Figure 10:**
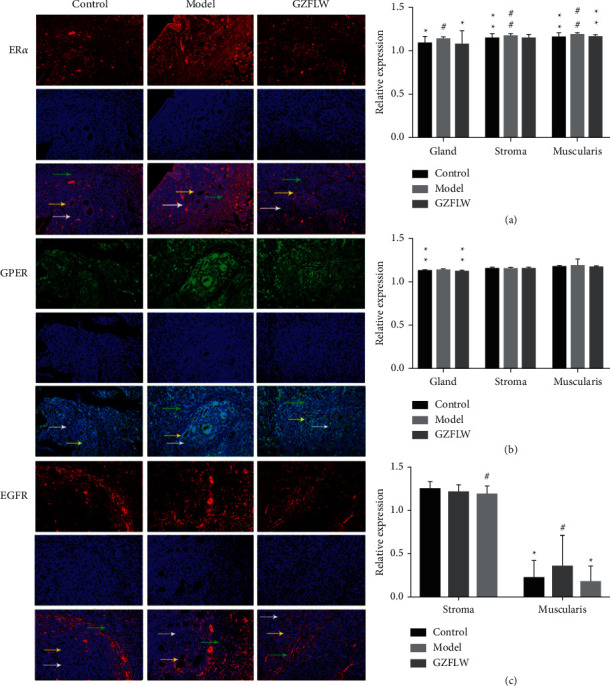
Immunofluorescence staining of ER*α*, GPER1, and EGFR at the EMI (×200). (a) ER*α* expression in glandular, stromal, and muscle layer of EMI. (b) GPER expression in glandular, stromal, and muscle layer of EMI. (c) EGFR expression in the stromal and muscle layer of EMI. The cells indicated by yellow arrow represent gland, the cells indicated by white arrow represent stroma, and the cells indicated by green arrow represent muscularis. Compared with the control group, ^#^*P* < 0.05 and  ^##^*P* < 0.01; compared with the model group,  ^*∗*^*P* < 0.05 and   ^*∗∗*^*P* < 0.01.

**Figure 11:**
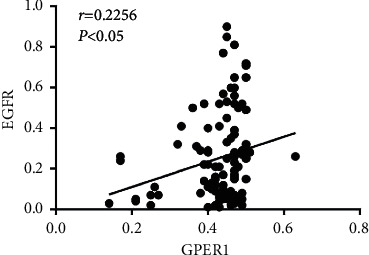
Correlation analysis of GPER1 and EGFR expression.

The immunofluorescence assay results showed that ER*α* and GPER were expressed in glandular epithelial cells, stromal cells, and muscularis cells. EGFR was expressed in stromal cells, muscularis cells, and some glandular epithelial cells (Figure 10). Compared with the control group, the expression of ER*α* in the gland, stroma, and muscularis of the model group was significantly enhanced; after GZFLW treatment, the expression of ER*α* in the gland and muscularis was obviously decreased and there was no significant difference between the control group and GZFLW group. Compared with the control group, the expression of GPER in the gland was markedly enhanced in the model group; after GZFLW treatment, the expression was considerably decreased. The expression of EGFR in the muscularis was significantly higher in the model group when compared to the control group, and the expression of EGFR in the muscularis of GZFLW group was significantly lower compared to that in the control group. Moreover, the expressions of GPER and EGFR in the muscle layer were positively correlated (Figure 11).

## 4. Discussion

Due to AM's complex pathophysiology, a totally resolving cure is yet to be found. The efficacy of TCM in treating AM has been confirmed through several hundred years' practices and experimental research [11]. Previous studies have found that TCM treatment can alleviate dysmenorrhea, shorten the menstrual time, decrease menstrual volume, reduce uterine volume, and lower CA125 levels in AM patients [12–14]. GZFLW is one of the classic traditional Chinese herbal formulas widely used in the clinical treatment of AM [8], and studies have confirmed that the side effect of GZFLW on AM is only 1.99% [15]. We found that the oral treatment of GZFLW exhibited a therapy effect on AM mice by suppressing the depth of invasion without disruption to the normal sexual cycle.

As a hormone-dependent disease, the disorder of estrogen and its receptor is known to promote and exacerbate AM [16, 17]. It has been reported that AM is usually accompanied by a high level of estrogen [17], and the critical enzyme expression of estrogen metabolism regulation in ectopic endometrial stromal cells is significantly increased [18]. Targeting estrogen has excellent potential in the therapy of AM [19, 20]. In our study, 9 out of 55 compounds from GZFLW may act as estrogen regulators, including beta-sitosterol, hederagenin, quercetin, paeoniflorin, kaempferol, taxifolin, pachymic acid, ellagic acid, and baicalein. Quercetin and baicalin are flavonoids derived from GZFLW component herbs RPR. Both of them can inhibit the proliferation and migration, consequently depress the invasion of AM ectopic endometrial stromal cells, and significantly alleviate pain allergy symptoms [21–23]. Kaempferol and beta-sitosterol, derived from GZFLW component herbs including CM, CT, RPR, and PK, have been shown to have antiproliferative effects on mice models of endometriosis diseases [24–26]. The effect of reducing estrogen-dependent abnormal proliferation has been demonstrated to be mediated via a downregulation of ER*α* [21, 27–29]. Paeoniflorin can downregulate the ER*α* level of AM primary cells [30]. Several active components, including taxifolin, beta-sitosterol, baicalein, and hederagenin, were validated as the potential antiestrogen compounds in GZFLW by molecular docking because of their high binding ability with ER*α*. The animal experiments found that ER*α* expression in AM mice model was significantly upregulated in glandular epithelial cells, stromal cells, and myometrial cells. It is acknowledged that endocrine regulatory treatments, such as oral contraceptives, oral and nonoral progesterone, danazol, and gonadotropin-releasing hormone (GnRH) analogs, have been used to control menstrual pain and menorrhagia by inhibiting uterine ER*α* expression. Still, they interfere with the menstrual cycle of AM women [31]. Those therapies lead to prolonged diestrus stages and even persistent diestrus in AM mice [32], which was associated with the cycle lengthening and disorder [33]. Our data showed that the ER*α* expression decreased and the pathological infiltration depth reduced in GZFLW group, while the diestrus/metestrus stages time was not prolonged. The result suggested that the treatment of TCM did not interfere with the estrous cycle in mice, which may be attributed to the selective action of the GZFLW on the local lesion.

Of note, the endocrine resistance pathway and EGFR tyrosine kinase signaling pathway may also contribute to the therapeutic mechanism of GZFLW against AM predicted by the network pharmacology analysis. Indeed, endocrine resistance is a complex pathophysiological process, known to lead to unresponsiveness of the regular endocrine therapy in a particular proportion of AM patients. It was reported that the EGFR gene polymorphism is involved in the endometriosis's susceptibility [34]. EGF/EGFR is of importance to the migration and invasion ability of endometrial stromal cells (ESC) [35], the reduction of which would inhibit the proliferation and vitality of AM ESC [36]. Subsequently, the results based on the molecular docking showed that the main compounds of GZFLW had a good binding ability with EGFR. In line with this observation, studies have shown that GZFLW can reduce the level of EGF in rats, thereby inhibiting the abnormal proliferation [37]. It has been proved that quercetin [38] can lower blood concentrations of EGF. Baicalein [39] and taxifolin [40] have also been reported to inhibit EGFR signaling by repressing EGFR-Tyr1068 autophosphorylation and then interfere with EGFR associated downstream signaling transduction. The results of our animal experiments showed that EGFR was upregulated in the model group compared to the control group and remarkably decreased after GZFLW treatment, which confirmed that EGF/EGFR is a potential target of GZFLW in treating AM. Besides, we proved there was a positive correlation between the expressions of GPER and EGFR, which may contribute to endocrine resistance. Indeed, endocrine resistance is a complex pathophysiological process, known to lead to unresponsiveness of the regular endocrine therapy in a particular proportion of AM patients [41]. In addition to ER deletion mutation, the EGF tyrosine kinase signaling pathway activated via GPER is another important cause of acquired estrogen tolerance. Previous studies have found that the gene polymorphism of GPER affects the risk of AM [42], and the expression of GPER is significantly increased in AM [43]. GPER can promote heparin-binding EGF release from the cell surface via activating Src family tyrosine kinases, and the activation of the EGF/EGFR signaling pathway eventually leads to epithelial-mesenchymal transition in AM [44]. In addition to the epithelial cells of the uterine gland, a similar trend was observed in the myometrium. Myometrium has the ability of stem cell-like abnormal differentiation [45]. Considering that the metaplasia of uterine smooth muscle cells was regarded as one crucial reason for the occurrence and development of AM [46], we speculate that GZFLW leads to the downregulation of the GPER/EGFR signaling pathway, which subsequently suppresses the estrogen resistance of smooth muscle cells and inhibits the abnormal differentiation of them.

In conclusion, based on the results of network pharmacology and animal experiments, we think that GZFLW can synergistically inhibit the abnormal proliferation and differentiation of AM via downregulating the ER*α* estrogen signaling pathway and suppress estrogen resistance of myocytes. This effect has no apparent interference in the normal endocrine.

## 5. Conclusion

There are several limitations in our study. Firstly, the databases we used can help predict the potential mechanism of GZFLW in treating AM. Nevertheless, most of them are based on the current research results, limiting the discovery of new therapeutic targets. Moreover, the primary detection indexes of the animal experiment only include ER*α*, GPER1, and EGFR, and this is not enough and comprehensive for data analysis. Finally, the number of animals is relatively small and may lead to study biases. Although network pharmacology strategy could help researchers simplify the complex system of TCM formulas and offer a novel research strategy for studying the mechanism of other TCM formulas, the mechanism remains confirmed by further experiments.

## Figures and Tables

**Table 1:** Information on 89 active ingredients.

Herb name	Mol ID	Compound	Code name	OB/%	DL/%
Cinnamon Twig	MOL001736	(-)-Taxifolin	CT-1	60.51	0.27
	MOL004576	Taxifolin	CT-2	57.84	0.27
	MOL000492	(+)-Catechin	CT-3	54.83	0.24
	MOL000073	Epicatechin	CT-4	48.96	0.24
	MOL000358	*β*-Sitosterol	CT-5	36.91	0.75
	MOL000359	Sitosterol	CT-6	36.91	0.75
	MOL000991	Cinnamaldehyde	CT-7	31.99	0.02
	MOL002295	Cinnamic acid	CT-8	19.68	0.03
	MOL000219	Benzoic acid	CT-9	31.55	0.02

*Poria cocos*	MOL000282	Ergosta-7,22-dien-3*β*-ol	PC-1	43.51	0.72
	MOL000283	Ergosterol peroxide	PC-2	40.36	0.81
	MOL000275	Trametenolic acid	PC-3	38.71	0.8
	MOL000296	Hederagenin	PC-4	36.91	0.75
	MOL000289	Pachymic acid	PC-5	33.63	0.81

Cortex Moutan	MOL000211	Mairin	CM-1	55.38	0.78
	MOL000492	(+)-Catechin	CM-2	54.83	0.24
	MOL000098	Quercetin	CM-3	46.43	0.28
	MOL000422	Kaempferol	CM-4	41.88	0.24
	MOL000359	Sitosterol	CM-5	36.91	0.75
	MOL000874	Paeonol	CM-6	28.79	0.04
	MOL000513	Gallic acid	CM-7	31.69	0.04

Radix Paeoniae Rubra	MOL001918	Paeoniflorgenone	RPR-1	87.59	0.37
	MOL006992	(2R,3 R)-4-Methoxyl-distylin	RPR-2	59.98	0.3
	MOL000492	(+)-Catechin	RPR-3	54.83	0.24
	MOL001924	Paeoniflorin	RPR-4	53.87	0.79
	MOL000449	Stigmasterol	RPR-5	43.83	0.76
	MOL001002	Ellagic acid	RPR-6	43.06	0.43
	MOL004355	Spinasterol	RPR-7	42.98	0.76
	MOL002776	Baicalin	RPR-8	40.12	0.75
	MOL005043	Campest-5-en-3*β*-ol	RPR-9	37.58	0.71
	MOL006999	Stigmast-7-en-3-ol	RPR-10	37.42	0.75
	MOL000358	*β*-Sitosterol	RPR-11	36.91	0.75
	MOL000359	Sitosterol	RPR-12	36.91	0.75
	MOL002714	Baicalein	RPR-13	33.52	0.21
	MOL002883	Ethyl oleate	RPR-14	32.4	0.19
	MOL000513	Gallic acid	RPR-15	31.69	0.04
	MOL000219	Benzoic acid	RPR-16	31.55	0.02

Peach Kernel	MOL001351	Gibberellin A44	PK-1	101.61	0.54
	MOL001353	Gibberellin A60	PK-2	93.17	0.53
	MOL001349	4a-Formyl-7*α*-hydroxy-1-methyl-8-methylidene-4a*α*,4b*β*-gibbane-1*α*,10*β*-dicarboxylic acid	PK-3	88.6	0.46
	MOL001344	Gibberellin A122-isolactone	PK-4	88.11	0.54
	MOL001329	2,3-Didehydro gibberellin A77	PK-5	88.08	0.53
	MOL001360	Gibberellin A77	PK-6	87.89	0.53
	MOL001340	Gibberellin A120	PK-7	84.85	0.45
	MOL001339	Gibberellin A119	PK-8	76.36	0.49
	MOL001358	Gibberellin 7	PK-9	73.8	0.5
	MOL001342	Gibberellin A121-isolactone	PK-10	72.7	0.54
	MOL001361	Gibberellin A87	PK-11	68.85	0.57
	MOL001355	Gibberellin A63	PK-12	65.54	0.54
	MOL001352	Gibberellin A54	PK-13	64.21	0.53
	MOL001328	2,3-Didehydro-gibberellin A70	PK-14	63.29	0.5
	MOL001323	Sitosterol *α*1	PK-15	43.28	0.78
	MOL001368	3-O-p-Coumaroylquinic acid	PK-16	37.63	0.29
	MOL000493	Campesterol	PK-17	37.58	0.71
	MOL000296	Hederagenin	PK-18	36.91	0.75
	MOL000358	*β*-Sitosterol	PK-19	36.91	0.75
	MOL001320	Amygdalin	PK-20	4.42	0.61

**Table 2:** Information of molecular docking.

	Taxifolin	Quercetin	Paeoniflorin	Beta-sitosterol	Baicalein	Hederagenin	Cinnamic acid
EGFR	−6.93	−5.3	−3.91	−8.26	−5.85	−7.72	−5.43
ER*α*	−5.87	−4.85	−3.94	−6.59	−6.45	−7.06	−4.95

## Data Availability

The underlying data used to support the findings of this study are included within the supplementary information file.

## Supplementary Materials

Supplementary Materials Table S1: detailed information of GZFLW compounds. Table S2: detailed information on therapeutic targets of AM. Table S3: detailed information of PPI network of 48 overlapped targets. Table S4: KEGG enrichment of overlapped targets from Metascape Database.
