# Supplementary material for: Integrating Network Pharmacology and Experimental Validation Deciphers the Mechanism of Guizhi Fuling Wan against Adenomyosis
Source: Evid Based Complement Alternat Med. 2021 Oct 26;2021:6034147. doi: 10.1155/2021/6034147 (PMC8563128; doi:10.1155/2021/6034147)
Supplement: Supplementary Materials — Table S1: detailed information of GZFLW compounds. Table S2: detailed information on therapeutic targets of AM. Table S3: detailed information of PPI network of 48 overlapped targets. Table S4: KEGG enrichment of overlapped targets from Metascape Database. [file 6034147.f1.zip › 6034147.f1/Table S3.pdf]

#node1	node2	node1_string_id	node2_string_id
AKT1	HMOX1	9606.ENSP00000451828	9606.ENSP00000216117
AKT1	NFKBIA	9606.ENSP00000451828	9606.ENSP00000216797
AKT1	MMP2	9606.ENSP00000451828	9606.ENSP00000219070
AKT1	CCL2	9606.ENSP00000451828	9606.ENSP00000225831
AKT1	CCND1	9606.ENSP00000451828	9606.ENSP00000227507
AKT1	CAT	9606.ENSP00000451828	9606.ENSP00000241052
AKT1	EGF	9606.ENSP00000451828	9606.ENSP00000265171
AKT1	TP53	9606.ENSP00000451828	9606.ENSP00000269305
AKT1	ERBB2	9606.ENSP00000451828	9606.ENSP00000269571
AKT1	NOTCH1	9606.ENSP00000451828	9606.ENSP00000277541
AKT1	GJA1	9606.ENSP00000451828	9606.ENSP00000282561
AKT1	BAX	9606.ENSP00000451828	9606.ENSP00000293288
AKT1	NOS3	9606.ENSP00000451828	9606.ENSP00000297494
AKT1	SLC2A4	9606.ENSP00000451828	9606.ENSP00000320935
AKT1	CAV1	9606.ENSP00000451828	9606.ENSP00000339191
AKT1	ESR2	9606.ENSP00000451828	9606.ENSP00000343925
AKT1	PTGS2	9606.ENSP00000451828	9606.ENSP00000356438
AKT1	PTEN	9606.ENSP00000451828	9606.ENSP00000361021
AKT1	MMP9	9606.ENSP00000451828	9606.ENSP00000361405
AKT1	NFE2L2	9606.ENSP00000451828	9606.ENSP00000380252
AKT1	BCL2	9606.ENSP00000451828	9606.ENSP00000381185
AKT1	CDKN1A	9606.ENSP00000451828	9606.ENSP00000384849
AKT1	IL6	9606.ENSP00000451828	9606.ENSP00000385675
AKT1	IGF2	9606.ENSP00000451828	9606.ENSP00000391826
AKT1	ESR1	9606.ENSP00000451828	9606.ENSP00000405330
AKT1	IL10	9606.ENSP00000451828	9606.ENSP00000412237
AKT1	CDKN2A	9606.ENSP00000451828	9606.ENSP00000418915
AKT1	RXRA	9606.ENSP00000451828	9606.ENSP00000419692
AKT1	HIF1A	9606.ENSP00000451828	9606.ENSP00000437955
AKT1	ITGB3	9606.ENSP00000451828	9606.ENSP00000452786
AKT1	VEGFA	9606.ENSP00000451828	9606.ENSP00000478570
BAX	TP53	9606.ENSP00000293288	9606.ENSP00000269305
BAX	BCL2	9606.ENSP00000293288	9606.ENSP00000381185
BCL2	TP53	9606.ENSP00000381185	9606.ENSP00000269305
BCL2	RXRA	9606.ENSP00000381185	9606.ENSP00000419692
BCL2	ESR1	9606.ENSP00000381185	9606.ENSP00000405330
BIRC5	TP53	9606.ENSP00000301633	9606.ENSP00000269305
CAT	HMOX1	9606.ENSP00000241052	9606.ENSP00000216117
CAT	PTGS2	9606.ENSP00000241052	9606.ENSP00000356438
CAT	XDH	9606.ENSP00000241052	9606.ENSP00000368727
CAT	IL6	9606.ENSP00000241052	9606.ENSP00000385675
CAT	NFE2L2	9606.ENSP00000241052	9606.ENSP00000380252
CAT	NOS3	9606.ENSP00000241052	9606.ENSP00000297494
CAV1	CCND1	9606.ENSP00000339191	9606.ENSP00000227507
CAV1	EGF	9606.ENSP00000339191	9606.ENSP00000265171
CAV1	TP53	9606.ENSP00000339191	9606.ENSP00000269305
CAV1	ERBB2	9606.ENSP00000339191	9606.ENSP00000269571
CAV1	EGFR	9606.ENSP00000339191	9606.ENSP00000275493
CAV1	PPARG	9606.ENSP00000339191	9606.ENSP00000287820
CAV1	NOS3	9606.ENSP00000339191	9606.ENSP00000297494
CAV1	PTEN	9606.ENSP00000339191	9606.ENSP00000361021
CAV1	VEGFA	9606.ENSP00000339191	9606.ENSP00000478570
CAV1	PTGS2	9606.ENSP00000339191	9606.ENSP00000356438
CAV1	ESR1	9606.ENSP00000339191	9606.ENSP00000405330
CCL2	HMOX1	9606.ENSP00000225831	9606.ENSP00000216117
CCL2	MMP2	9606.ENSP00000225831	9606.ENSP00000219070
CCL2	TP53	9606.ENSP00000225831	9606.ENSP00000269305

CCL2	IFNG	9606.ENSP00000225831	9606.ENSP00000229135
CCL2	MMP9	9606.ENSP00000225831	9606.ENSP00000361405
CCL2	PTGS2	9606.ENSP00000225831	9606.ENSP00000356438
CCL2	MMP3	9606.ENSP00000225831	9606.ENSP00000299855
CCL2	MMP1	9606.ENSP00000225831	9606.ENSP00000322788
CCL2	VEGFA	9606.ENSP00000225831	9606.ENSP00000478570
CCL2	IL1B	9606.ENSP00000225831	9606.ENSP00000263341
CCL2	IL6	9606.ENSP00000225831	9606.ENSP00000385675
CCL2	IL10	9606.ENSP00000225831	9606.ENSP00000412237
CCND1	MMP2	9606.ENSP00000227507	9606.ENSP00000219070
CCND1	ESR2	9606.ENSP00000227507	9606.ENSP00000343925
CCND1	MMP9	9606.ENSP00000227507	9606.ENSP00000361405
CCND1	VEGFA	9606.ENSP00000227507	9606.ENSP00000478570
CCND1	IL6	9606.ENSP00000227507	9606.ENSP00000385675
CCND1	EGF	9606.ENSP00000227507	9606.ENSP00000265171
CCND1	ERBB2	9606.ENSP00000227507	9606.ENSP00000269571
CCND1	PGR	9606.ENSP00000227507	9606.ENSP00000325120
CCND1	PTEN	9606.ENSP00000227507	9606.ENSP00000361021
CCND1	IGF2	9606.ENSP00000227507	9606.ENSP00000391826
CCND1	EGFR	9606.ENSP00000227507	9606.ENSP00000275493
CCND1	TP53	9606.ENSP00000227507	9606.ENSP00000269305
CCND1	NOTCH1	9606.ENSP00000227507	9606.ENSP00000277541
CCND1	ESR1	9606.ENSP00000227507	9606.ENSP00000405330
CCND1	CDKN2A	9606.ENSP00000227507	9606.ENSP00000418915
CCND1	CDKN1A	9606.ENSP00000227507	9606.ENSP00000384849
CDKN1A	TP53	9606.ENSP00000384849	9606.ENSP00000269305
CDKN1A	ESR1	9606.ENSP00000384849	9606.ENSP00000405330
CDKN1A	HIF1A	9606.ENSP00000384849	9606.ENSP00000437955
CDKN1A	CDKN2A	9606.ENSP00000384849	9606.ENSP00000418915
CDKN2A	TP53	9606.ENSP00000418915	9606.ENSP00000269305
CDKN2A	ERBB2	9606.ENSP00000418915	9606.ENSP00000269571
CDKN2A	EGFR	9606.ENSP00000418915	9606.ENSP00000275493
CDKN2A	PTEN	9606.ENSP00000418915	9606.ENSP00000361021
CDKN2A	IL6	9606.ENSP00000418915	9606.ENSP00000385675
CDKN2A	VEGFA	9606.ENSP00000418915	9606.ENSP00000478570
CDKN2A	HIF1A	9606.ENSP00000418915	9606.ENSP00000437955
COMT	CYP1A1	9606.ENSP00000354511	9606.ENSP00000369050
CYP19A1	ERBB2	9606.ENSP00000379683	9606.ENSP00000269571
CYP19A1	PGR	9606.ENSP00000379683	9606.ENSP00000325120
CYP19A1	ESR2	9606.ENSP00000379683	9606.ENSP00000343925
CYP19A1	CYP1A1	9606.ENSP00000379683	9606.ENSP00000369050
CYP19A1	ESR1	9606.ENSP00000379683	9606.ENSP00000405330
CYP1A1	TP53	9606.ENSP00000369050	9606.ENSP00000269305
CYP1A1	GSTM1	9606.ENSP00000369050	9606.ENSP00000311469
CYP1A1	NCOA2	9606.ENSP00000369050	9606.ENSP00000399968
CYP1A1	RXRA	9606.ENSP00000369050	9606.ENSP00000419692
EGF	MMP2	9606.ENSP00000265171	9606.ENSP00000219070
EGF	IL1B	9606.ENSP00000265171	9606.ENSP00000263341
EGF	NOS3	9606.ENSP00000265171	9606.ENSP00000297494
EGF	PTEN	9606.ENSP00000265171	9606.ENSP00000361021
EGF	TP53	9606.ENSP00000265171	9606.ENSP00000269305
EGF	MMP1	9606.ENSP00000265171	9606.ENSP00000322788
EGF	IL10	9606.ENSP00000265171	9606.ENSP00000412237
EGF	GJA1	9606.ENSP00000265171	9606.ENSP00000282561
EGF	PGR	9606.ENSP00000265171	9606.ENSP00000325120
EGF	ESR1	9606.ENSP00000265171	9606.ENSP00000405330
EGF	IL6	9606.ENSP00000265171	9606.ENSP00000385675
EGF	PTGS2	9606.ENSP00000265171	9606.ENSP00000356438

EGF	MMP9	9606.ENSP00000265171	9606.ENSP00000361405
EGF	IGF2	9606.ENSP00000265171	9606.ENSP00000391826
EGF	VEGFA	9606.ENSP00000265171	9606.ENSP00000478570
EGF	ERBB2	9606.ENSP00000265171	9606.ENSP00000269571
EGF	EGFR	9606.ENSP00000265171	9606.ENSP00000275493
EGFR	IFNG	9606.ENSP00000275493	9606.ENSP00000229135
EGFR	TP53	9606.ENSP00000275493	9606.ENSP00000269305
EGFR	ERBB2	9606.ENSP00000275493	9606.ENSP00000269571
EGFR	ESR2	9606.ENSP00000275493	9606.ENSP00000343925
EGFR	PPARG	9606.ENSP00000275493	9606.ENSP00000287820
EGFR	PGR	9606.ENSP00000275493	9606.ENSP00000325120
EGFR	MMP9	9606.ENSP00000275493	9606.ENSP00000361405
EGFR	IGF2	9606.ENSP00000275493	9606.ENSP00000391826
EGFR	PTGS2	9606.ENSP00000275493	9606.ENSP00000356438
EGFR	IL10	9606.ENSP00000275493	9606.ENSP00000412237
EGFR	NOTCH1	9606.ENSP00000275493	9606.ENSP00000277541
EGFR	IL6	9606.ENSP00000275493	9606.ENSP00000385675
EGFR	ESR1	9606.ENSP00000275493	9606.ENSP00000405330
EGFR	MMP3	9606.ENSP00000275493	9606.ENSP00000299855
EGFR	PTEN	9606.ENSP00000275493	9606.ENSP00000361021
EGFR	HIF1A	9606.ENSP00000275493	9606.ENSP00000437955
EGFR	VEGFA	9606.ENSP00000275493	9606.ENSP00000478570
ERBB2	MMP2	9606.ENSP00000269571	9606.ENSP00000219070
ERBB2	TP53	9606.ENSP00000269571	9606.ENSP00000269305
ERBB2	MMP1	9606.ENSP00000269571	9606.ENSP00000322788
ERBB2	MMP9	9606.ENSP00000269571	9606.ENSP00000361405
ERBB2	IGF2	9606.ENSP00000269571	9606.ENSP00000391826
ERBB2	PTGS2	9606.ENSP00000269571	9606.ENSP00000356438
ERBB2	NOTCH1	9606.ENSP00000269571	9606.ENSP00000277541
ERBB2	PTEN	9606.ENSP00000269571	9606.ENSP00000361021
ERBB2	ESR1	9606.ENSP00000269571	9606.ENSP00000405330
ERBB2	PGR	9606.ENSP00000269571	9606.ENSP00000325120
ERBB2	VEGFA	9606.ENSP00000269571	9606.ENSP00000478570
ERBB2	IL6	9606.ENSP00000269571	9606.ENSP00000385675
ESR1	TP53	9606.ENSP00000405330	9606.ENSP00000269305
ESR1	NOS3	9606.ENSP00000405330	9606.ENSP00000297494
ESR1	PGR	9606.ENSP00000405330	9606.ENSP00000325120
ESR1	ESR2	9606.ENSP00000405330	9606.ENSP00000343925
ESR1	PTGS2	9606.ENSP00000405330	9606.ENSP00000356438
ESR1	PTEN	9606.ENSP00000405330	9606.ENSP00000361021
ESR1	IL6	9606.ENSP00000405330	9606.ENSP00000385675
ESR1	NCOA2	9606.ENSP00000405330	9606.ENSP00000399968
ESR1	HIF1A	9606.ENSP00000405330	9606.ENSP00000437955
ESR1	VEGFA	9606.ENSP00000405330	9606.ENSP00000478570
ESR2	NOS3	9606.ENSP00000343925	9606.ENSP00000297494
ESR2	NCOA2	9606.ENSP00000343925	9606.ENSP00000399968
GJA1	VEGFA	9606.ENSP00000282561	9606.ENSP00000478570
HIF1A	HMOX1	9606.ENSP00000437955	9606.ENSP00000216117
HIF1A	MMP2	9606.ENSP00000437955	9606.ENSP00000219070
HIF1A	TP53	9606.ENSP00000437955	9606.ENSP00000269305
HIF1A	NOTCH1	9606.ENSP00000437955	9606.ENSP00000277541
HIF1A	NOS3	9606.ENSP00000437955	9606.ENSP00000297494
HIF1A	PTEN	9606.ENSP00000437955	9606.ENSP00000361021
HIF1A	VEGFA	9606.ENSP00000437955	9606.ENSP00000478570
HMOX1	PPARG	9606.ENSP00000216117	9606.ENSP00000287820
HMOX1	MMP9	9606.ENSP00000216117	9606.ENSP00000361405
HMOX1	TP53	9606.ENSP00000216117	9606.ENSP00000269305
HMOX1	IL1B	9606.ENSP00000216117	9606.ENSP00000263341

HMOX1	PTGS2	9606.ENSP00000216117	9606.ENSP00000356438
HMOX1	IL6	9606.ENSP00000216117	9606.ENSP00000385675
HMOX1	NOS3	9606.ENSP00000216117	9606.ENSP00000297494
HMOX1	VEGFA	9606.ENSP00000216117	9606.ENSP00000478570
HMOX1	IL10	9606.ENSP00000216117	9606.ENSP00000412237
HMOX1	NFE2L2	9606.ENSP00000216117	9606.ENSP00000380252
IFNG	MMP9	9606.ENSP00000229135	9606.ENSP00000361405
IFNG	IL1B	9606.ENSP00000229135	9606.ENSP00000263341
IFNG	IL6	9606.ENSP00000229135	9606.ENSP00000385675
IFNG	IL10	9606.ENSP00000229135	9606.ENSP00000412237
IGF2	MMP2	9606.ENSP00000391826	9606.ENSP00000219070
IGF2	TP53	9606.ENSP00000391826	9606.ENSP00000269305
IGF2	MMP1	9606.ENSP00000391826	9606.ENSP00000322788
IGF2	PTEN	9606.ENSP00000391826	9606.ENSP00000361021
IGF2	VEGFA	9606.ENSP00000391826	9606.ENSP00000478570
IL10	IL1B	9606.ENSP00000412237	9606.ENSP00000263341
IL10	NOS3	9606.ENSP00000412237	9606.ENSP00000297494
IL10	PTGS2	9606.ENSP00000412237	9606.ENSP00000356438
IL10	PTEN	9606.ENSP00000412237	9606.ENSP00000361021
IL10	MMP9	9606.ENSP00000412237	9606.ENSP00000361405
IL10	IL6	9606.ENSP00000412237	9606.ENSP00000385675
IL10	VEGFA	9606.ENSP00000412237	9606.ENSP00000478570
IL1B	NFKBIA	9606.ENSP00000263341	9606.ENSP00000216797
IL1B	MMP2	9606.ENSP00000263341	9606.ENSP00000219070
IL1B	NOS3	9606.ENSP00000263341	9606.ENSP00000297494
IL1B	PPARG	9606.ENSP00000263341	9606.ENSP00000287820
IL1B	MMP1	9606.ENSP00000263341	9606.ENSP00000322788
IL1B	MMP3	9606.ENSP00000263341	9606.ENSP00000299855
IL1B	VEGFA	9606.ENSP00000263341	9606.ENSP00000478570
IL1B	MMP9	9606.ENSP00000263341	9606.ENSP00000361405
IL1B	PTGS2	9606.ENSP00000263341	9606.ENSP00000356438
IL1B	IL6	9606.ENSP00000263341	9606.ENSP00000385675
IL6	MMP2	9606.ENSP00000385675	9606.ENSP00000219070
IL6	TP53	9606.ENSP00000385675	9606.ENSP00000269305
IL6	NOTCH1	9606.ENSP00000385675	9606.ENSP00000277541
IL6	PPARG	9606.ENSP00000385675	9606.ENSP00000287820
IL6	NOS3	9606.ENSP00000385675	9606.ENSP00000297494
IL6	MMP3	9606.ENSP00000385675	9606.ENSP00000299855
IL6	SLC2A4	9606.ENSP00000385675	9606.ENSP00000320935
IL6	MMP1	9606.ENSP00000385675	9606.ENSP00000322788
IL6	PTGS2	9606.ENSP00000385675	9606.ENSP00000356438
IL6	MMP9	9606.ENSP00000385675	9606.ENSP00000361405
IL6	VEGFA	9606.ENSP00000385675	9606.ENSP00000478570
ITGB3	VEGFA	9606.ENSP00000452786	9606.ENSP00000478570
MMP1	MMP2	9606.ENSP00000322788	9606.ENSP00000219070
MMP1	TP53	9606.ENSP00000322788	9606.ENSP00000269305
MMP1	MMP3	9606.ENSP00000322788	9606.ENSP00000299855
MMP1	VEGFA	9606.ENSP00000322788	9606.ENSP00000478570
MMP1	MMP9	9606.ENSP00000322788	9606.ENSP00000361405
MMP2	PTGS2	9606.ENSP00000219070	9606.ENSP00000356438
MMP2	TP53	9606.ENSP00000219070	9606.ENSP00000269305
MMP2	MMP9	9606.ENSP00000219070	9606.ENSP00000361405
MMP2	MMP3	9606.ENSP00000219070	9606.ENSP00000299855
MMP2	VEGFA	9606.ENSP00000219070	9606.ENSP00000478570
MMP3	PTGS2	9606.ENSP00000299855	9606.ENSP00000356438
MMP3	MMP9	9606.ENSP00000299855	9606.ENSP00000361405
MMP9	TP53	9606.ENSP00000361405	9606.ENSP00000269305
MMP9	NOTCH1	9606.ENSP00000361405	9606.ENSP00000277541

MMP9	PPARG	9606.ENSP00000361405	9606.ENSP00000287820
MMP9	NOS3	9606.ENSP00000361405	9606.ENSP00000297494
MMP9	PTGS2	9606.ENSP00000361405	9606.ENSP00000356438
MMP9	PTEN	9606.ENSP00000361405	9606.ENSP00000361021
MMP9	VEGFA	9606.ENSP00000361405	9606.ENSP00000478570
NCOA2	PPARG	9606.ENSP00000399968	9606.ENSP00000287820
NCOA2	SLC2A4	9606.ENSP00000399968	9606.ENSP00000320935
NCOA2	PGR	9606.ENSP00000399968	9606.ENSP00000325120
NCOA2	RXRA	9606.ENSP00000399968	9606.ENSP00000419692
NFE2L2	TP53	9606.ENSP00000380252	9606.ENSP00000269305
NFE2L2	PPARG	9606.ENSP00000380252	9606.ENSP00000287820
NFKBIA	TP53	9606.ENSP00000216797	9606.ENSP00000269305
NFKBIA	PTGS2	9606.ENSP00000216797	9606.ENSP00000356438
NFKBIA	RXRA	9606.ENSP00000216797	9606.ENSP00000419692
NOS3	TP53	9606.ENSP00000297494	9606.ENSP00000269305
NOS3	PPARG	9606.ENSP00000297494	9606.ENSP00000287820
NOS3	PTGS2	9606.ENSP00000297494	9606.ENSP00000356438
NOS3	VEGFA	9606.ENSP00000297494	9606.ENSP00000478570
NOTCH1	TP53	9606.ENSP00000277541	9606.ENSP00000269305
NOTCH1	VEGFA	9606.ENSP00000277541	9606.ENSP00000478570
NOTCH1	PTEN	9606.ENSP00000277541	9606.ENSP00000361021
PGR	TP53	9606.ENSP00000325120	9606.ENSP00000269305
PGR	PTEN	9606.ENSP00000325120	9606.ENSP00000361021
PPARG	TP53	9606.ENSP00000287820	9606.ENSP00000269305
PPARG	VEGFA	9606.ENSP00000287820	9606.ENSP00000478570
PPARG	PTEN	9606.ENSP00000287820	9606.ENSP00000361021
PPARG	PTGS2	9606.ENSP00000287820	9606.ENSP00000356438
PPARG	SLC2A4	9606.ENSP00000287820	9606.ENSP00000320935
PPARG	RXRA	9606.ENSP00000287820	9606.ENSP00000419692
PTEN	TP53	9606.ENSP00000361021	9606.ENSP00000269305
PTEN	VEGFA	9606.ENSP00000361021	9606.ENSP00000478570
PTGER3	PTGS2	9606.ENSP00000349003	9606.ENSP00000356438
PTGS2	TP53	9606.ENSP00000356438	9606.ENSP00000269305
PTGS2	VEGFA	9606.ENSP00000356438	9606.ENSP00000478570
RXRA	SLC2A4	9606.ENSP00000419692	9606.ENSP00000320935
TP53	VEGFA	9606.ENSP00000269305	9606.ENSP00000478570

neighborhood_on_chromosome	gene_fusion	phylogenetic_cooccurrence	homology	coexpression
0	0	0	0	0
0	0	0	0	0.061
0	0	0	0	0.061
0	0	0	0	0
0	0	0	0	0.061
0.043	0	0	0	0
0	0	0	0	0
0	0	0	0	0.054
0	0	0	0.556	0.053
0	0	0	0	0.077
0	0	0	0	0
0	0	0	0	0.061
0	0	0	0	0.049
0	0	0	0	0
0	0	0	0	0
0	0	0	0	0
0	0	0	0	0.062
0	0	0	0	0.06
0	0	0	0	0.061
0	0	0	0	0
0	0	0	0	0.061
0	0	0	0	0
0	0	0	0	0.055
0	0	0	0	0
0	0	0	0	0.055
0	0	0	0	0
0	0	0	0	0.061
0	0	0	0	0.085
0	0	0	0	0
0	0	0	0	0.08
0	0	0	0	0.063
0	0	0	0	0.05
0	0	0	0.645	0
0	0	0	0	0.05
0	0	0	0	0
0	0	0	0	0.069
0	0	0	0	0.088
0	0	0	0	0.062
0	0	0	0	0
0	0	0	0	0.062
0	0	0	0	0
0	0	0	0	0
0	0	0	0	0.041
0	0	0	0	0.214
0	0	0	0	0
0	0	0	0	0
0	0	0	0	0.06
0	0	0	0	0.335
0	0	0	0	0.071
0	0	0	0	0
0	0	0	0	0
0	0	0	0	0.076
0	0	0	0	0.084
0	0	0	0	0
0	0	0	0	0.094
0	0	0	0	0.155
0	0	0	0	0

0	0	0	0	0.063
0	0	0	0	0.076
0	0	0	0	0.117
0	0	0	0	0.107
0	0	0	0	0.102
0	0	0	0	0
0	0	0	0	0.126
0	0	0	0	0.267
0	0	0	0	0
0	0	0	0	0.139
0	0	0	0	0
0	0	0	0	0
0	0	0	0	0.069
0	0	0	0	0
0	0	0	0	0
0	0	0	0	0.098
0	0	0	0	0
0	0	0	0	0
0	0	0	0	0.092
0	0	0	0	0.2
0	0	0	0	0.061
0	0	0	0	0.057
0	0	0	0	0
0	0	0	0	0.061
0	0	0	0	0.084
0	0	0	0	0
0	0	0	0	0
0	0	0	0	0
0	0	0	0	0.081
0	0	0	0	0
0	0	0	0	0
0	0	0	0	0
0	0	0	0	0.062
0	0	0	0	0
0	0	0	0	0
0	0	0	0	0
0	0	0	0	0.061
0	0	0	0	0
0	0	0	0	0
0	0	0	0	0.062
0	0	0.409	0.563	0
0	0	0	0	0
0	0	0	0	0
0	0	0	0	0.061
0	0	0	0	0.062
0	0	0	0	0.051
0	0	0	0	0
0	0	0	0	0
0	0	0	0	0
0	0	0	0	0.067
0	0	0	0	0
0	0	0	0	0
0	0	0	0	0
0	0	0	0	0.061
0	0	0	0	0
0	0	0	0	0
0	0	0	0	0
0	0	0	0	0.061

0	0	0	0	0
0	0	0	0	0
0	0	0	0	0
0	0	0	0	0.16
0	0	0	0	0.16
0	0	0	0	0
0	0	0	0	0
0	0	0	0.941	0.077
0	0	0	0	0
0	0	0	0	0.062
0	0	0	0	0
0	0	0	0	0
0	0	0	0	0.088
0	0	0	0	0.061
0	0	0	0	0
0	0	0	0	0
0	0	0	0	0
0	0	0	0	0
0	0	0	0	0.076
0	0	0	0	0
0	0	0	0	0
0	0	0	0	0.089
0	0	0	0	0.062
0	0	0	0	0
0	0	0	0	0
0	0	0	0	0
0	0	0	0	0.061
0	0	0	0	0.061
0	0	0	0	0
0	0	0	0	0
0	0	0	0	0
0	0	0	0	0
0	0	0	0	0
0	0	0	0	0
0	0	0	0	0
0	0	0	0	0
0	0	0	0.679	0.102
0	0	0	0.925	0.061
0	0	0	0	0
0	0	0	0	0
0	0	0	0	0
0	0	0	0	0.062
0	0	0	0	0
0	0	0	0	0.061
0	0	0	0	0
0	0	0	0	0
0	0	0	0	0
0	0	0	0	0
0	0	0	0	0
0	0	0	0	0
0	0	0	0	0
0	0	0	0	0
0	0	0	0	0.061
0	0	0	0	0.062
0	0	0	0	0
0	0	0	0	0.088
0	0	0	0	0.061
0	0	0	0	0.07

0	0	0	0	0.076
0	0	0	0	0.073
0	0	0	0	0
0	0	0	0	0
0	0	0	0	0
0	0	0	0	0
0	0	0	0	0
0	0	0	0	0.082
0	0	0	0	0.064
0	0	0	0	0.064
0	0	0	0	0.165
0	0	0	0	0
0	0	0	0	0.076
0	0	0	0	0
0	0	0	0	0.069
0	0	0	0	0.085
0	0	0	0	0
0	0	0	0	0.061
0	0	0	0	0
0	0	0	0	0
0	0	0	0	0.077
0	0	0	0	0
0	0	0	0	0.155
0	0	0	0	0
0	0	0	0	0.062
0	0	0	0	0.061
0	0	0	0	0.128
0	0	0	0	0.107
0	0	0	0	0
0	0	0	0	0.2
0	0	0	0	0.561
0	0	0	0	0.43
0	0	0	0	0.076
0	0	0	0	0.062
0	0	0	0	0
0	0	0	0	0
0	0	0	0	0.058
0	0	0	0	0.112
0	0	0	0	0
0	0	0	0	0.154
0	0	0	0	0.314
0	0	0	0	0.074
0	0	0	0	0.063
0	0	0	0	0
0	0	0	0.869	0.154
0	0	0	0	0
0	0	0.298	0.953	0.529
0	0	0	0	0.069
0	0	0	0.75	0.518
0	0	0	0	0.066
0	0	0	0	0.062
0	0	0	0.929	0.061
0	0	0	0.88	0.181
0	0	0	0	0.065
0	0	0	0	0.095
0	0	0	0.791	0.518
0	0	0	0	0
0	0	0	0	0.069

0	0	0	0	0
0	0	0	0	0.062
0	0	0	0	0.097
0	0	0	0	0
0	0	0	0	0
0	0	0	0	0.062
0	0	0	0	0
0	0	0	0	0
0	0	0	0	0.061
0	0	0	0	0
0	0	0	0	0
0	0	0	0	0
0	0	0	0	0.076
0	0	0	0	0
0	0	0	0	0
0	0	0	0	0.053
0	0	0	0	0
0	0	0	0	0.062
0	0	0	0	0
0	0	0	0	0
0	0	0	0	0.14
0	0	0	0	0.062
0	0	0	0	0
0	0	0	0	0
0	0	0	0	0
0	0	0	0	0.061
0	0	0	0	0
0	0	0	0	0.061
0	0	0	0.694	0.061
0	0	0	0	0
0	0	0	0	0
0	0	0	0	0
0	0	0	0	0
0	0	0	0	0.082
0	0	0	0	0.061
0	0	0	0	0

experimentally_determined_interaction	database_annotated	automated_textmining	combined_score
0.379	0	0.828	0.889
0.433	0	0.586	0.76
0	0	0.833	0.836
0	0	0.794	0.794
0	0	0.941	0.943
0.073	0.9	0.715	0.971
0.05	0	0.932	0.933
0.147	0.9	0.941	0.994
0.543	0	0.943	0.737
0.438	0	0.874	0.929
0.053	0	0.815	0.818
0.379	0	0.581	0.734
0.693	0.9	0.97	0.999
0.261	0	0.902	0.925
0.057	0.9	0.815	0.981
0.407	0	0.722	0.828
0.058	0	0.848	0.854
0.472	0	0.981	0.99
0	0	0.885	0.887
0.344	0	0.786	0.854
0	0.9	0.426	0.941
0.696	0.9	0.851	0.995
0	0	0.886	0.888
0.262	0	0.794	0.841
0.407	0.9	0.867	0.991
0.079	0	0.821	0.828
0.214	0	0.707	0.764
0.085	0.9	0.271	0.93
0.362	0.9	0.827	0.988
0.34	0.9	0.425	0.96
0.342	0	0.95	0.966
0.578	0.9	0.669	0.984
0.58	0.9	0.734	0.967
0.525	0.9	0.705	0.984
0.064	0.9	0.046	0.902
0.064	0.9	0.244	0.925
0	0.9	0.571	0.957
0.194	0	0.883	0.904
0.131	0	0.685	0.715
0	0	0.71	0.717
0	0	0.735	0.735
0	0	0.743	0.743
0	0	0.745	0.745
0	0	0.697	0.752
0	0.9	0.746	0.973
0	0	0.746	0.746
0.379	0	0.705	0.813
0.393	0.9	0.844	0.992
0.466	0	0.473	0.716
0.379	0.9	0.915	0.994
0.32	0	0.671	0.766
0	0	0.772	0.78
0.379	0	0.669	0.795
0.4	0	0.734	0.834
0	0	0.81	0.821
0	0	0.802	0.825
0	0	0.725	0.725

0	0	0.794	0.798
0	0	0.843	0.849
0	0	0.847	0.859
0.379	0	0.795	0.876
0.379	0	0.806	0.882
0	0	0.914	0.914
0	0	0.945	0.95
0	0	0.977	0.982
0	0.9	0.955	0.995
0	0	0.676	0.709
0.05	0	0.706	0.709
0	0	0.765	0.765
0.17	0	0.743	0.784
0	0	0.812	0.813
0	0	0.844	0.844
0.43	0	0.799	0.888
0.43	0	0.817	0.892
0	0	0.893	0.893
0	0	0.901	0.907
0.157	0	0.886	0.917
0.379	0	0.942	0.963
0	0.9	0.824	0.982
0.384	0.9	0.858	0.99
0.313	0.9	0.901	0.992
0.993	0.9	0.857	0.999
0.696	0.9	0.97	0.999
0.379	0	0.559	0.714
0	0.9	0.399	0.937
0	0.9	0.815	0.981
0.384	0.9	0.968	0.997
0.176	0	0.813	0.839
0.176	0	0.745	0.78
0.23	0	0.88	0.906
0	0	0.752	0.752
0	0	0.761	0.761
0.379	0.9	0.402	0.959
0	0.9	0.677	0.967
0	0	0.789	0.789
0.085	0	0.851	0.857
0.085	0	0.845	0.855
0	0.9	0.68	0.941
0.085	0	0.96	0.962
0.074	0	0.745	0.754
0.077	0.65	0.892	0.962
0	0.9	0.224	0.92
0.085	0.9	0.212	0.922
0	0	0.797	0.798
0	0	0.743	0.743
0	0	0.719	0.719
0.084	0	0.729	0.749
0	0	0.758	0.758
0	0	0.77	0.771
0	0	0.796	0.796
0	0	0.803	0.807
0	0	0.823	0.823
0	0	0.836	0.836
0	0	0.844	0.844
0.082	0	0.856	0.866

0	0	0.871	0.871
0	0.9	0.672	0.965
0	0.9	0.937	0.993
0.406	0.9	0.94	0.996
0.933	0.9	0.972	0.999
0	0.6	0.44	0.766
0.294	0	0.951	0.964
0.699	0.9	0.967	0.971
0.077	0	0.739	0.749
0.402	0	0.65	0.787
0.077	0	0.804	0.811
0	0	0.828	0.828
0	0.6	0.579	0.833
0	0	0.869	0.872
0	0.6	0.713	0.88
0.157	0	0.887	0.901
0	0.6	0.811	0.921
0.402	0	0.908	0.943
0	0.9	0.543	0.954
0.472	0	0.94	0.967
0	0.9	0.743	0.973
0	0.6	0.947	0.979
0	0	0.752	0.758
0.167	0	0.937	0.945
0	0	0.721	0.721
0	0	0.78	0.781
0	0.6	0.511	0.8
0.305	0	0.75	0.822
0.157	0	0.823	0.844
0.185	0	0.884	0.902
0.32	0	0.926	0.947
0.36	0	0.931	0.954
0	0.6	0.895	0.956
0	0.9	0.739	0.972
0.379	0	0.919	0.948
0.472	0.9	0.749	0.985
0.472	0.9	0.964	0.964
0.379	0.9	0.967	0.941
0.05	0	0.771	0.773
0.393	0	0.686	0.801
0	0	0.769	0.769
0.9	0.9	0.583	0.995
0.413	0	0.695	0.813
0	0	0.813	0.816
0.091	0.9	0.671	0.967
0.48	0.9	0.5	0.971
0	0	0.755	0.755
0	0	0.729	0.73
0	0	0.72	0.721
0.379	0.9	0.674	0.977
0.379	0.9	0.517	0.967
0	0.9	0.706	0.969
0	0	0.751	0.757
0.348	0.9	0.947	0.996
0	0	0.735	0.735
0	0	0.745	0.759
0	0	0.761	0.766
0	0	0.814	0.819

0	0	0.827	0.834
0	0	0.841	0.847
0.185	0	0.829	0.855
0	0	0.867	0.867
0	0	0.867	0.868
0	0.9	0.968	0.996
0	0	0.735	0.735
0	0	0.898	0.902
0	0	0.937	0.939
0	0	0.944	0.946
0	0.9	0.399	0.945
0	0	0.777	0.777
0	0.9	0.257	0.925
0	0	0.743	0.743
0	0.9	0.809	0.98
0	0.9	0.96	0.996
0	0	0.708	0.709
0	0	0.831	0.836
0	0	0.734	0.735
0	0	0.831	0.831
0	0.9	0.974	0.997
0	0	0.884	0.885
0	0	0.833	0.853
0.379	0	0.802	0.872
0	0	0.723	0.729
0	0	0.789	0.794
0	0	0.845	0.859
0	0	0.862	0.872
0	0	0.877	0.877
0	0	0.895	0.913
0	0	0.943	0.974
0	0	0.973	0.984
0	0	0.816	0.823
0	0	0.864	0.867
0	0	0.832	0.832
0	0	0.812	0.812
0	0	0.796	0.8
0	0	0.747	0.766
0	0	0.765	0.766
0	0	0.814	0.836
0	0	0.922	0.945
0	0	0.92	0.923
0	0	0.957	0.959
0.064	0.9	0.421	0.941
0	0.9	0.921	0.922
0	0	0.707	0.707
0	0.9	0.921	0.953
0	0	0.827	0.832
0	0.9	0.91	0.961
0	0	0.696	0.704
0	0	0.797	0.802
0	0.9	0.958	0.908
0	0.9	0.892	0.923
0	0.8	0.899	0.979
0	0	0.774	0.787
0	0.9	0.911	0.959
0	0	0.8	0.8
0.076	0	0.747	0.764

0	0	0.709	0.709
0	0	0.778	0.783
0	0	0.725	0.741
0	0	0.735	0.735
0	0.8	0.946	0.989
0.974	0.9	0.356	0.998
0	0.9	0.097	0.905
0.461	0	0.499	0.718
0.963	0.9	0.458	0.997
0.099	0	0.79	0.803
0.379	0	0.685	0.796
0.454	0	0.515	0.723
0	0	0.794	0.801
0	0.9	0.185	0.915
0	0	0.731	0.731
0.091	0	0.741	0.757
0.261	0	0.786	0.835
0	0.9	0.934	0.993
0	0	0.916	0.917
0	0	0.864	0.864
0.087	0	0.945	0.953
0	0	0.789	0.793
0.064	0	0.776	0.781
0	0.9	0.751	0.974
0	0	0.769	0.769
0.064	0	0.775	0.785
0.05	0	0.817	0.819
0	0.9	0.736	0.973
0.961	0.9	0.821	0.997
0.472	0.9	0.963	0.997
0	0	0.839	0.839
0.056	0	0.821	0.824
0.379	0	0.869	0.915
0	0	0.914	0.918
0	0.9	0.198	0.918
0	0	0.918	0.918
