# Supplementary material for: Integrating Network Pharmacology and Experimental Validation Deciphers the Mechanism of Guizhi Fuling Wan against Adenomyosis
Source: Evid Based Complement Alternat Med. 2021 Oct 26;2021:6034147. doi: 10.1155/2021/6034147 (PMC8563128; doi:10.1155/2021/6034147)
Supplement: Supplementary Materials — Table S1: detailed information of GZFLW compounds. Table S2: detailed information on therapeutic targets of AM. Table S3: detailed information of PPI network of 48 overlapped targets. Table S4: KEGG enrichment of overlapped targets from Metascape Database. [file 6034147.f1.zip › 6034147.f1/10.1-Integrating network pharmacology and experimental validation deciphers the mechanism of Guizhi Fuling Wan against adenomyosis (1) (1).doc]

**Evidence-Based Complementary and Alternative Medicine**

**Integrating network pharmacology and experimental validation deciphers the mechanism of Guizhi Fuling Wan against adenomyosis**

Haoxian Wang,^1^ Jihong Zhang,^2^ Qinqin Zhu,^1^ Xianyun Fu,^1^ Chenjie Li^2^

^1^ Medical College, China Three Gorges University, Yichang 443002, China.

^2^ The Second People’s Hospital of China Three Gorges University, Yichang 443000, China.

Correspondence: Xianyun Fu; dinnar1@163.com

Correspondence: Chenjie Li; 179073543@qq.com

**Abstract**

**Aim** This study aimed to predict the key targets and endocrine mechanisms of Guizhi Fuling Wan (GZFLW) in treating adenomyosis (AM) through network pharmacology, molecular docking, and animal experiment verification. **Methods** The related ingredients and targets of GZFLW in treating AM were screened out using TCMSP, BATMAN-TCM, SwissTargetPrediction, and PubChem Database. Then, the protein-protein interaction (PPI) analysis and the network of compound-hub targets were constructed. At the same time, the key targets were uploaded to the Metascape Database for KEGG pathway enrichment analysis. After that, the molecular docking technology of the main active components and hub targets was performed. What’s more, the high-performance liquid chromatography (HPLC) analysis and mid-infrared spectroscopy analysis were established to screen and verify the potential active compounds. Furthermore, animal experiments were used to verify the results of network pharmacology analysis. **Results** A total of 55 active ingredients of GZFLW and 44 overlapping targets of GZFLW in treating AM were obtained. After screening, 25 hub targets were collected, including ESR1, EGF, and EGFR. Then, the KEGG pathway enrichment analysis results indicated that the endocrine therapeutic mechanism of GZFLW against AM is mainly associated with the estrogen signaling pathway, endocrine resistance, and an EGFR tyrosine kinase signaling pathway. Then, molecular docking showed that the significant compounds of GZFLW had a strong binding ability with ERα and EGFR. And the HPLC analysis and mid-infrared spectroscopy analysis verified some active ingredients of GZFLW. More importantly, the animal experiments confirmed that the GZFLW could down-regulate the abnormal infiltration of the endometrial epithelium into the myometrium and had no interference with the normal sexual cycle. This effect may be directly related to intervening the local estrogen signaling pathway of the endometrial myometrial interface (EMI). It may also be associated with the myometrium cells' estrogen resistance via GPER/EGFR signaling pathway. **Conclusion** The endocrine mechanism of GZFLW in treating AM was explored based on network pharmacology, molecular docking, and animal experiments, which provided a theoretical basis for the clinical application of GZFLW.

**Keywords:** Guizhi Fuling Wan; network pharmacology; molecular docking; adenomyosis; estrogen receptor

# Introduction

# In 2020, 1.03% of the women were suffering from adenomyosis (AM), while approximately 90% of them were accompanied by menorrhagia, progressive dysmenorrhea, pelvic pain, dysmenorrheal, and infertility.^1^ AM is characterized by the progressive invasion of endometrial cells into the myometrium, attributed to endocrine disorders, chronic hypoxia, inflammation in the endometrial myometrial interface (EMI). Current therapeutic approaches, including operative treatment, non-steroidal anti-inflammatory drugs, and endocrine therapy, have been used to suppress inflammation, relieve pain, and improve fertility. However, several limitations include poor patients’ response, high prices, and long-term side-effects. Therefore, it is of great clinical significance to develop alternative therapeutic strategies to improve the outcome of AM patients.

Traditional Chinese medicine (TCM), which has been widely used in clinical practice in China for thousands of years, has made considerable contributions to the relief of AM.^6^ Guizhi Fuling Wan (GZFLW) is an effective Chinese medicinal formula for AM that is used in TCM. This medication consists of the following five herbs: Cinnamon Twig (CT), Poria cocos (PC), Cortex Moutan (CM), Radix Paeoniae Rubra (RPR), and Peach Kernel (PK). The complex composition of GZFLW makes it is not easy to conduct mechanistic studies on it. Thus, the“multi-component and multi-target” therapeutic mechanism of GZFLW in treating AM is still obscure, hindering its clinical use. Hence, for a comprehensive understanding of the mechanism of action of GZFLW, a novel analysis method is needed.^9^

In this study, a network pharmacology strategy was adopted for investigating the mechanism of GZFLW against AM. We screened 55 representative compounds in five herbs of GZFLW. Their potential targets in treating AM were identified. Furthermore, molecular docking technology was used to verify the binding ability of active ingredients to several potential targets. In addition, animal experiments have further confirmed some of the hypotheses. The results revealed that GZFLW might modulate the estrogen signaling pathway, endocrine resistance, EGFR tyrosine kinase signaling pathway to exert an effect. These results may provide novel sights into the anti-AM effect of GZFLW.

**Material and methods**

**Chemical ingredients database building**

The active ingredients of the five herbs contained in GZFLW were obtained using the Traditional Chinese Medicine Systems Pharmacology Database (TCMSP, <http://lsp.nwu.edu.cn/tcmsp.php>) and the Bioinformatics Analysis Tool for Molecular Mechanism of Traditional Chinese Medicine (BATMAN-TCM, <http://bionet.ncpsb.org/batman-tcm/index.php>), which are both bioinformatics analysis tools for the main components of TCM.

**Active ingredients screening**

The oral bioavailability (OB) and drug-likeness (DL) were used as screening indexes. OB is the percentage of oral drugs absorbed into the systemic circulation, which is a frequently-used pharmacokinetic parameter. DL refers to the property of measuring the absorption, distribution, metabolism, and excretion (ADME) of drug molecules, which could help optimize pharmacokinetic and pharmaceutical properties. The OB and DL indices of all the related ingredients are presented in the TCMSP. We set the indexes of OB ≥ 30% and DL ≥ 0.18 as the screening criteria of active ingredients. Briefly, components that satisfied the screening threshold of OB and DL were regarded as the candidate compounds.

**GZFLW targets prediction**

The targets of active ingredients were searched in TCMSP, BATMAN-TCM, SwissTargetPrediction Database ([http://www.swisstarget](http://www.swisstarget/)prediction.ch/index.php) and PubChem Database (<https://pubchem.ncbi.nlm.nih.gov/>), with the species limited as ‘‘Homo sapiens’’.

**Adenomyosis therapeutic targets prediction**

Targets associated with AM were collected by GeneCards Database (<https://www.genecards.org/>) and Online Mendelian Inheritance in Man Database (OMIM, [https://www.omim.org](https://www.omim.org/)). We searched these databases with the keyword “adenomyosis” to obtain related targets. After deletion of duplications, candidate targets against AM were identified by matching the targets of the active ingredients of GZFLW and the genes related to AM. These targets were then uploaded to STRING Database (https://string-db.rog/) to obtain the protein-protein interaction (PPI) information between two proteins or multiple proteins with the confidence ≥ 0.7.

**Network topological feature set definitions**

For each node in the interaction network, we selected two parameters to evaluate its topological features: “Degree” reflects the number of other nodes interacting with this node; the percentage of all shortest paths measures “Betweenness Centrality” (BC). Nodes greater than or equal to the median are screened as hub nodes for GZFLW against AM.

**KEGG enrichment analysis**

We used the Metascape Database (<http://metascape.org/>) to obtain the potential pathways of GZFLW in treating AM. The *P* ≤ 0.05 was regarded as the cutoff value.

**Molecular docking of hub targets and active ingredients**

Molecular docking is a computational tool to predict the binding ability and connection type of proteins and ligands. It can calculate and predict the conformation and directions of ligands at active protein sites. AutoDock 1.5.6 (http://autodock.scripps.edu/) was a molecular docking software for network pharmacology based prediction and analysis, which could be used to dock the hub targets and active ingredients. In this process, structures files in the formats of 3D of hub targets we chose was obtained from RCSB Protein Data Bank (PDB, <http://www.rcsb.org/>): EGFR (PDB ID: 2GS2), ERα (PDB ID: 6CHW); structures files in the formats of 3D of active compounds were provided from ZINC Database (http://zinc.docking.org/) and PubChem Database (<https://pubchem.ncbi.nlm.nih.gov/>). During the docking process, the PDBQT files of these structures were used in AutoDock software, as these files contained the information of potential ligands and receptors required for the process. After that, the Lamarckian genetic algorithm was used to obtain docking results and the binding energy of ligands and receptors. At last, the results were output in .dlg files and uploaded to PyMOL software for establishing the 3D results binding models. Binding energy was used as a docking score to evaluate the protein-ligand binding potential of active ingredients to hub targets. Among them, those results with value≤-5 were selected and considered to have moderate binding potential and tight combination.

**HPLC and Mid-infrared spectroscopy analysis**

The HPLC finger printing of CT, RPR, PK, and CM in GZFLW was constructed using Series LC-2010A Liquid Chromatograph (Shimadzu, Japan). Use octadecyl silane bonded silica as the stationary phase (column: hypersil ODS C18 4.6mm×250mm, 5μm). The mobile phase was ammonium acetate (A); water (B). The flow rate was kept constant at 1 mL/min. The system operated at 30 °C and the injection volume was 10 μL (CT, RPR, PK) and 5 μL (CM). The detection wavelength was respectively kept at 285 nm (CT), 230 nm (RPR), 274 nm (CM) and 210 nm (PK). The reference substances (cinnamic acid, cinnamaldehyde, gallic aicd, catechin, paeonol, paeoniflorin, amygdalin) were prepared by Chengdu Ruifensi Biotechnology Co., Ltd (Chengdu, China).

The active ingredients of PC were analyzed by mid-infrared spectroscopy. The ingredient was weighed, adding about 100mg KBr powder which was dried at 120 ℃ for 4H. Then, grind and mix well in the mortar, transfer to the mold, and press the samples into transparent slices at a pressure of about 30 MPa for 1 ~ 2 min. The sample slice thickness was about 1 ~ 2 mm. The Fourier transform infrared spectrometer (Deuterated triglycine sulfate detector) was used with a spectral range of 4000 ~ 400 cm^– 1^. Each sample was repeated twice.

**In vivo experiment**

Virgin female ICR mice (26–29g, 7 weeks old) were provided by the Experimental Animals Center of China Three Gorges University. All the animals were housed in a temperature of 22±2°C, humidity of 55–65% and light controlled (12h light/ 12h dark cycle) vivarium, with food and water ad libitum. Animal welfare and experimental procedures were carried out in accordance with the Guide for the Care and Use of Laboratory Animals, and were approved by the China Three Gorges University Medical Animal Ethics Commission (Approval NO. 20190801).

Twenty five mice were implanted with a single pituitary gland each, under the capsule of the right uterus at 7 weeks of age and then were divided randomly into 2 groups: model group (n=14), GZFLW group (n=11). After intraperitoneally anesthetizing with emulsion (Xian Nippon Pharmaceutical Co. Ltd. Xian, China, No.H19990282, 100 mg/kg), pituitary obtained from age-matched male mice was grafted into the right uterine lumen in female mice. Then, the gentamycin solution (0.25 ml, 20000 units/20 g) was dropped intraperitoneally to prevent infection. Mice receiving no pituitary grafts served as controls (n=8). One day after the pituitary grafting, 11 mice were intragastriced with decocting-free granules of GZFLE (4.25mg/ g of body Weight, consisting of CT, PC, CM, RPR, and PK at the Ratio of 1:1:1:1:1, Beijing Kangrentang pharmaceutical Co. Ltd., Beijing, China, NO 19026231, 19027291, 19019661, 19004891, 19006412), dissolved in aqueous solution, every day for 8 weeks (GZFLW group ). The remaining 22 mice (model group 14, control group 8) were intragastriced with the same dosage of saline as a control vehicle. All mice were killed at 15 weeks of age by decapitation, after the estrous cycle had been checked by vaginal smear.

Haematoxylin and eosin (HE) staining were used to observe the depth of invasion. Immunofluorescence was adopted to identify the expressions of ERα, GPER, and EGFR in the parts of glandular, stromal, and muscular in the endometrial myometrial interface (EMI) (magnification, 200×, every microscope field taking three different parts to analysis).

**Data and statistical analysis**

Data were analyzed using software of SPSS 20 and presented as mean ± standard deviation (SD).. Statistical differences between groups were evaluated by one-way analysis of variance (ANOVA). The Chi-square test was applied to analyze the categorical variables. Pearson analysis was used for correlation. *P* < 0.05 was regarded as a statistically significant difference.

# Results and Discussion

**Active ingredients and targets of GZFLW**

By retrieving from the databases, there were 498 related ingredients of GZFLW in total, including 222 ingredients in CT, 33 ingredients in PC, 56 ingredients in CM, 121 ingredients in RPR, and 66 ingredients in PK. . After eliminating the redundancy and screening with OB and DL, 55 active components and 364 corresponding targets of GZFLW were obtained. It is worth noting that cinnamaldehyde (OB = 31.99%, DL = 0.02), cinnamic acid (OB = 19.68%, DL = 0.03), benzoic acid (OB = 31.55%, DL = 0.02), paeonol (OB = 28.79%, DL = 0.04), gallic acid (OB = 31.69, DL = 0.04), and amygdalin (OB = 4.42%, DL = 0,61) have a relatively low OB or DL value, but they were included in this study because they were the vital pharmacological compounds identified, which were confirmed to the previous study (Table 1) (Table S1).

Table 1: Information on 89 active ingredients.

| Herb name | Mol ID | compound | Code name | OB/% | DL/% |
| --- | --- | --- | --- | --- | --- |
| Cinnamon Twig | MOL001736 | (-)-taxifolin | CT-1 | 60.51 | 0.27 |
|  | MOL004576 | [taxifolin](http://tcmspw.com/molecule.php?qn=4576) | CT-2 | 57.84 | 0.27 |
|  | MOL000492 | [(+)-catechin](http://tcmspw.com/molecule.php?qn=492) | CT-3 | 54.83 | 0.24 |
|  | MOL000073 | [epicatechin](http://tcmspw.com/molecule.php?qn=73) | CT-4 | 48.96 | 0.24 |
|  | MOL000358 | [β-sitosterol](http://tcmspw.com/molecule.php?qn=358) | CT-5 | 36.91 | 0.75 |
|  | MOL000359  MOL000991  MOL002295  MOL000219 | [sitosterol](http://tcmspw.com/molecule.php?qn=359)  cinnamaldehyde  cinnamic acid  benzoic acid | CT-6  CT-7  CT-8  CT-9 | 36.91  31.99  19.68  31.55 | 0.75  0.02  0.03  0.02 |
| Poria Cocos | MOL000282 | [ergosta-7,22-dien-3β-ol](http://tcmspw.com/molecule.php?qn=282) | PC-1 | 43.51 | 0.72 |
|  | MOL000283 | [ergosterol peroxide](http://tcmspw.com/molecule.php?qn=283) | PC-2 | 40.36 | 0.81 |
|  | MOL000275 | [trametenolic acid](http://tcmspw.com/molecule.php?qn=275) | PC-3 | 38.71 | 0.8 |
|  | MOL000296  MOL000289 | [hederagenin](http://tcmspw.com/molecule.php?qn=296)  pachymic acid | PC-4  PC-5 | 36.91  33.63 | 0.75  0.81 |
| [Cortex Moutan](http://tcmspw.com/tcmspsearch.php?qr=Cortex Moutan&qsr=herb_en_name&token=b24793da79e8f6a6110e0b954b5d9475) | MOL000211 | [mairin](http://tcmspw.com/molecule.php?qn=211) | CM-1 | 55.38 | 0.78 |
|  | MOL000492 | [(+)-catechin](http://tcmspw.com/molecule.php?qn=492) | CM-2 | 54.83 | 0.24 |
|  | MOL000098 | [quercetin](http://tcmspw.com/molecule.php?qn=98) | CM-3 | 46.43 | 0.28 |
|  | MOL000422 | [kaempferol](http://tcmspw.com/molecule.php?qn=422) | CM-4 | 41.88 | 0.24 |
|  | MOL000359  MOL000874  MOL000513 | [sitosterol](http://tcmspw.com/molecule.php?qn=359)  paeonol  gallic acid | CM-5  CM-6  CM-7 | 36.91  28.79  31.69 | 0.75  0.04  0.04 |
| [Radix Paeoniae Rubra](http://tcmspw.com/tcmspsearch.php?qr=Radix Paeoniae Rubra&qsr=herb_en_name&token=b24793da79e8f6a6110e0b954b5d9475) | MOL001918 | [paeoniflorgenone](http://tcmspw.com/molecule.php?qn=1918) | RPR-1 | 87.59 | 0.37 |
|  | MOL006992 | [(2R,3R)-4-methoxyl-distylin](http://tcmspw.com/molecule.php?qn=6992) | RPR-2 | 59.98 | 0.3 |
|  | MOL000492 | [(+)-catechin](http://tcmspw.com/molecule.php?qn=492) | RPR-3 | 54.83 | 0.24 |
|  | MOL001924 | [paeoniflorin](http://tcmspw.com/molecule.php?qn=1924) | RPR-4 | 53.87 | 0.79 |
|  | MOL000449 | [stigmasterol](http://tcmspw.com/molecule.php?qn=449) | RPR-5 | 43.83 | 0.76 |
|  | MOL001002 | [ellagic acid](http://tcmspw.com/molecule.php?qn=1002) | RPR-6 | 43.06 | 0.43 |
|  | MOL004355 | [spinasterol](http://tcmspw.com/molecule.php?qn=4355) | RPR-7 | 42.98 | 0.76 |
|  | MOL002776 | [baicalin](http://tcmspw.com/molecule.php?qn=2776) | RPR-8 | 40.12 | 0.75 |
|  | MOL005043 | [campest-5-en-3β-ol](http://tcmspw.com/molecule.php?qn=5043) | RPR-9 | 37.58 | 0.71 |
|  | MOL006999 | stigmast-7-en-3-ol | RPR-10 | 37.42 | 0.75 |
|  | MOL000358 | [β-sitosterol](http://tcmspw.com/molecule.php?qn=358) | RPR-11 | 36.91 | 0.75 |
|  | MOL000359 | [sitosterol](http://tcmspw.com/molecule.php?qn=359) | RPR-12 | 36.91 | 0.75 |
|  | MOL002714 | [baicalein](http://tcmspw.com/molecule.php?qn=2714) | RPR-13 | 33.52 | 0.21 |
|  | MOL002883  MOL000513  MOL000219 | [ethyl oleate](http://tcmspw.com/molecule.php?qn=2883)  gallic acid  benzoic acid | RPR-14  RPR-15  RPR-16 | 32.4  31.69  31.55 | 0.19  0.04  0.02 |
| Peach Kenel | MOL001351 | [gibberellin A44](http://tcmspw.com/molecule.php?qn=1351) | PK-1 | 101.61 | 0.54 |
|  | MOL001353 | [gibberellin A60](http://tcmspw.com/molecule.php?qn=1353) | PK-2 | 93.17 | 0.53 |
|  | MOL001349 | [4a-formyl-7α-hydroxy-1-methyl-8-methylidene-4aα,4bβ-gibbane-1α,10β-dicarboxylic acid](http://tcmspw.com/molecule.php?qn=1349) | PK-3 | 88.6 | 0.46 |
|  | MOL001344 | [gibberellin A122-isolactone](http://tcmspw.com/molecule.php?qn=1344) | PK-4 | 88.11 | 0.54 |
|  | MOL001329 | [2,3-didehydro gibberellin A77](http://tcmspw.com/molecule.php?qn=1329) | PK-5 | 88.08 | 0.53 |
|  | MOL001360 | [gibberellin A77](http://tcmspw.com/molecule.php?qn=1360) | PK-6 | 87.89 | 0.53 |
|  | MOL001340 | [gibberellin A120](http://tcmspw.com/molecule.php?qn=1340) | PK-7 | 84.85 | 0.45 |
|  | MOL001339 | [gibberellin A119](http://tcmspw.com/molecule.php?qn=1339) | PK-8 | 76.36 | 0.49 |
|  | MOL001358 | [gibberellin 7](http://tcmspw.com/molecule.php?qn=1358) | PK-9 | 73.8 | 0.5 |
|  | MOL001342 | [gibberellin A121-isolactone](http://tcmspw.com/molecule.php?qn=1342) | PK-10 | 72.7 | 0.54 |
|  | MOL001361 | [gibberellin A87](http://tcmspw.com/molecule.php?qn=1361) | PK-11 | 68.85 | 0.57 |
|  | MOL001355 | [giiberellin A63](http://tcmspw.com/molecule.php?qn=1355) | PK-12 | 65.54 | 0.54 |
|  | MOL001352 | [gibberellin A54](http://tcmspw.com/molecule.php?qn=1352) | PK-13 | 64.21 | 0.53 |
|  | MOL001328 | [2,3-didehydro gibberellin A70](http://tcmspw.com/molecule.php?qn=1328) | PK-14 | 63.29 | 0.5 |
|  | MOL001323 | [sitosterol α1](http://tcmspw.com/molecule.php?qn=1323) | PK-15 | 43.28 | 0.78 |
|  | MOL001368  MOL000493 | [3-O-p-Coumaroylquinic acid](http://tcmspw.com/molecule.php?qn=1368)  campesterol | PK-16  PK-17 | 37.63  37.58 | 0.29  0.71 |
|  | MOL000296 | [hederagenin](http://tcmspw.com/molecule.php?qn=296) | PK-18 | 36.91 | 0.75 |
|  | MOL000358  MOL001320 | [β-sitosterol](http://tcmspw.com/molecule.php?qn=358)  amygdalin | PK-19  PK-20 | 36.91  4.42 | 0.75  0.61 |

**Target analysis**

Via the keyword of "adenomyosis", the therapeutic targets of AM were obtained from GeneCards and OMIM Database with a total of 211 (Table S2). A total of 48 overlapping targets were then obtained as the related targets of GZFLW in treating AM (Figure 1).

From the STRING Database of "multiple proteins" interaction of targets, the data of the Protein-Protein Interaction (PPI) network of those 48 targets were subsequently obtained (Table S3). With confidence≥0.7, we gained 268 edges of 48 nodes. With betweenness≥14 and degree≥0.01, the topological analysis of targets mentioned above was performed. The results were 25 hub nodes with 183 edges, in which 25 nodes represented 25 central targets, including ESR1, EGF, and EGFR. When the 25 significant nodes and other 23 nodes were distributed with "degree" and "betweenness", the network of 48 nodes was constructed as Figure 2. The comprehensive network of "Herbs-Compounds-Hub targets" had 25 nodes related 26 ingredients, as determined by the Cytoscape 3.7.1 software (Figure 3).

Figure 1: The Venn diagram of the targets both in endometriosis targets and GZFLW targets.

Figure 2: The network of 48 nodes. The different color represents different nodes from each ingredients: The blue nodes represent the targets from CT, the pink nodes represent the targets from CM, the purple nodes represent the targets from RPR, the green nodes represent the targets from PC, and the yellow nodes represent the targets that are targeted by more than one ingredient. The node size is proportional to the target degree in the network. The edge color changes from light to dark reflect the betweenness value changes from low to high in the network.

Figure 3: Herbs-Compounds- Hub Targets Network. The triangle nodes represent herbs, square nodes represent active ingredients, and pink nodes represent key targets. RPR represents Radix Paeoniae Rubra, PC represents Poria Cocos, CT represents Cinnamon Twig, CM represents Cortex Moutan, and PK represents Peach Kenel. The node size is proportional to the target degree in the network.

**KEGG pathway enrichment analysis**

In the database of Metascape, we performed the KEGG pathway enrichment analysis on 48 targets and obtained 114 pathways (Table S4). We ranked these pathways according to the *P-*value of each enriched pathway in ascending order, and the top 20 pathways were selected, which mainly related to endocrine resistance, EGFR tyrosine kinase inhibitor resistance, and estrogen signaling pathway (Figure 4).

Figure 4: Information of 20 pathways about KEGG enrichment

**Molecular Docking**

To further explore the binding ability of active components and hub targets in GZFLW, molecular docking technology was used. Due to ERα and EGFR were the core targets of both estrogen pathway and endocrine resistance pathway, the two were selected to bind to active ingredients. 71.43% of the compounds (10/17) had binding energies lower than -5, which verified those active components may be well binding with ERα and EGFR (Table 2). The 3D binding mode of 2 major active ingredients in the active site of ERα and EGFR, respectively, is represented in Figure 5.

Figure 5: The network of molecular docking results. (a) EGFR-hederagenin. (b) ERα- hederagenin. (c) EGFR-β-sitosterol. (d) ERα-β-sitosterol. The yellow sticks represent EGFR and ERα. The blue sticks represent active compounds.

Table 2: Information of molecular docking.

|  | taxifolin | quercetin | paeoniflorin | beta-sitosterol | baicalein | hederagenin | cinnamic acid |
| --- | --- | --- | --- | --- | --- | --- | --- |
| EGFR  ERα | -6.93  -5.87 | -5.3  -4.85 | -3.91  -3.94 | -8.26  -6.59 | -5.85  -6.45 | -7.72  -7.06 | -5.43  -4.95 |

**HPLC analysis of CT, CM, RPR, PK**

There were six main peaks in the fingerprint of CT at 285 nm, and two active components were determined after comparison with standards; the ten main peaks in the fingerprint of CM at 274 nm were obtained, and three vital components were confirmed; there were five main peaks in the fingerprint of RPR at 230 nm, and among these, three active ingredients were determined; the results in the fingerprint of PK at 210 nm were four main peaks, defining one significant ingredient (Figure 6). All of the main peaks had good separation.

Figure 6: HPLC fingerprint of CT, CM, RPR, PK. (a) Cinnamon Twig (CT). (b) Cortex Moutan (CM). (c) Radix Paeoniae Rubra (RPR). (d) Peach Kenel (PK). 1: cinnamic acid; 2: cinnamaldehyde; 3: gallic aicd; 4: (+)-catechin; 5: paeonol; 6: paeoniflorin; 7: amygdalin.

**Mid-infrared spectroscopy**

Around 3400 cm^−1^, these frequencies was associated with O-H stretching. Around 2800 cm^-1^, the peaks of C-H antisymmetric stretching of methylene group were observed. Frequencies around 1600 cm^-1^ and 1450 cm^-1^ were associated with O-H and C-H bends. Below 1300 cm^-1^, the frequencies were associated with C-O stretching, that frequencies between 1200 and 1000 cm^-1^ showed the presence of polysaccharide. In addition, frequencies below 950 cm^-1^ were related to sugar ring skeleton stretching (Figure 7).

Figure 7: IR spectra of PC.

**Effect on infiltration depth in mice**

AM model was successfully established after pituitary grafts, and HE staining indicated that endometrial stroma and glandular epithelial cells infiltrated into the myometrium. According to the infiltration depth, the models can be divided into mild, moderate, and severe infiltration (Figure 8). The results showed that the proportion of moderate and severe infiltration decreased after GZFLW treatment.

Figure 8: HE staining of the transverse section of the uterus. (a) normal transverse section of the uterus. (b) mild infiltration. (c) moderate infiltration. (d) severe infiltration. Arrows indicate infiltration of endometrial glands and stroma into the inner myometrium (b), outer myometrium (c), and serosa (d).

**Effect on sexual cycle in mice**

The estrous cycle of mice can be divided into four stages: proestrus (mainly nucleated epithelial cells), estrus (mainly non-nucleated keratinocytes), metestrus (mainly a small number of keratinocytes and more leukocytes), and diestrus (mainly a large number of leukocytes). We divided the estrous cycles into two phases, combining metestrus with diestrus because of the leucocytic predominance.^10^ There is no significant difference in vaginal smears among the three groups by chi-square test, which confirmed that GZFLW treatment had no critical interference on the sexual cycle of mice (Figure 9).

Figure 9: Observation of vaginal smears and sexual cycle statistics of mice. (a) proestrus. (b) estrus. (c) metestrus. (d) diestrus.

**Immunofluorescence assay**

The immunofluorescence assay results showed that ERα and GPER were expressed in glandular epithelial cells, stromal cells, and muscularis cells. EGFR was expressed in stromal cells, muscularis cells, and some glandular epithelial cells (Figure 10). Compared with the control group, the expression of ERα in the gland, stroma, and muscularis of the model group was significantly enhanced; after GZFLW treatment, the expression of ERα in the gland, and muscularis was obviously decreased and there was no significant difference between the control group and GZFLW group. Compared with the control group, the expression of GPER in the gland was markedly enhanced in the model group; after GZFLW treatment, the expression was considerably decreased. The expression of EGFR in the muscularis was significantly higher in the model group when compared to the control, and the expression of EGFR in the muscularis of GZFLW group was significantly lower compared to the control group. Moreover, the expression of GPER and EGFR in the muscle layer was positively correlated (Figure 11).

Figure 10: Immunofluorescence staining of ERα，GPER1，EGFR at the EMI(×200). (a) ERα expression in gland, stroma and muscle layer of EMI. (b) GPER expression in glandular, stromal and muscle layer of EMI. (c) EGFR expression in the stroma and muscle layer of EMI. The cells indicated by yellow arrow represent gland, the cells indicated by white arrow represent stroma, and the cells indicated by green arrow represent muscularis. Compared with the control group, # *P* < 0.05, ##*P* < 0.01; compared with the model group, * *P* < 0.05, ** *P* < 0.01.

Figure 11: Correlation analysis of GPER1 and EGFR expression.

**Discussion**

Due to AM’s complex pathophysiology, a totally resolving cure is yet to be found. The efficacy of TCM in treating AM has been confirmed through several hundred years’ practices and experimental research.^11^ Previous studies have found that TCM treatment can alleviate dysmenorrhea, shorten the menstrual time, decrease menstrual volume, reduce uterine volume and lower CA125 levels in AM patients.^12-14^ GZFLW is one of the classic traditional Chinese herbal formulas widely used in the clinical treatment of AM,^8^ and studies have confirmed that the side effect of GZFLW on AM is only 1.99%.^15^ We found that the oral treatment of GZFLW exhibited a therapy effect on AM mice by suppressing the depth of invasion without disruption to the normal sexual cycle.

As a hormone-dependent disease, the disorder of estrogen and its receptor is known to promote and exacerbate AM. It has been reported that AM is usually accompanied by a high level of estrogen,^17^ and the critical enzyme expression of estrogen metabolism regulation in ectopic endometrial stromal cells is significantly increased.^18^ Targeting estrogen has excellent potential in the therapy of AM. In our study, 9 out of 55 compounds from GZFLW may act as estrogen regulators, including beta-sitosterol, hederagenin, quercetin, paeoniflorin, kaempferol, taxifolin, pachymic acid, ellagic acid, and baicalein. Quercetin and baicalin are flavonoids derived from GZFLW component herbs RPR. Both of them can inhibit the proliferation and migration, consequently depress the invasion of AM ectopic endometrial stromal cells, and significantly alleviate pain allergy symptoms.^21-23^ Kaempferol and beta-sitosterol, derived from GZFLW component herbs including CM, CT, RPR, and PK, have been shown to have anti-proliferative effects on mice models of endometriosis diseases.^24-26^ The effect of reducing estrogen-dependent abnormal proliferation has been demonstrated to be mediated via a downregulation of ERα. Paeoniflorin can down-regulate the ERα level of AM primary cells.^30^ Several active components, including taxifolin, beta-sitosterol, baicalein, hederagenin, were validated as the potential anti-estrogen compounds in GZFLW by molecular docking because of their high binding ability with ERα. The animal experiments found that ERα expression in AM mice model was significantly up-regulated in glandular epithelial cells, stromal cells, and myometrial cells. It is acknowledged that endocrine regulatory treatments, such as oral contraceptives, oral and non-oral progesterone, danazol, and gonadotropin-releasing hormone (GnRH) analogs have been used to control menstrual pain and menorrhagia by inhibiting uterine ERα expression. Still, they interfere with the menstrual cycle of AM women.^31^ Those therapies lead to prolonged diestrus stages, even persistent diestrus in AM mice,^32^ which was associated with the cycle lengthening and disorder.^33^ Our data showed that the ERα expression decreased and the pathological infiltration depth reduced.in GZFLW group while the diestrus/metestrus stages time was not prolonged. The result suggested that the treatment of TCM did not interfere with the estrous cycle in mice, which may be attributed to the selective action of the GZFLW on the local lesion.

Of note, the endocrine resistance pathway and EGFR tyrosine kinase signaling pathway may also contribute to the therapeutic mechanism of GZFLW against AM predicted by the network pharmacology analysis. Indeed, endocrine resistance is a complex pathophysiological process, known to lead to unresponsiveness of the regular endocrine therapy in a particular proportion of AM patients. It was reported that the EGFR gene polymorphism is involved in the endometriosis's susceptibility.^34^ EGF / EGFR is of importance to the migration and invasion ability of endometrial stromal cells (ESC),^35^ and the reduction of which would inhibit the proliferation and vitality of AM ESC.^36^ Subsequently, the results based on the molecular docking showed that the main compounds of GZFLW had a good binding ability with EGFR. In line with this observation, studies have shown that GZFLW can reduce the level of EGF in rats, thereby inhibiting the abnormal proliferation.^37^ It has been proved that quercetin^38^ can lowered blood concentrations of EGF. The baicalein^39^ and taxifolin^40^ have also been reported to inhibit EGFR signaling by repressing EGFR-Tyr1068 autophosphorylation, and then interfere with EGFR associated downstream signaling transduction. The results of our animal experiments showed that EGFR were up-regulated in the model group compared to the controls and remarkably decreased after GZFLW treatment, which confirmed that EGF/EGFR is a potential target of GZFLW in treating AM. Besides, we proved there was a positive correlation between the expression of GPER and EGFR, which may contributed to endocrine resistance. Indeed, endocrine resistance is a complex pathophysiological process, known to lead to unresponsiveness of the regular endocrine therapy in a particular proportion of AM patients.^41^ In addition to ER deletion mutation, the EGF tyrosine kinase signaling pathway activated via GPER is another important cause of acquired estrogen tolerance. Previous studies have found that the gene polymorphism of GPER affects the risk of AM,^42^ and the expression of GPER is significantly increased in AM.^43^ GPER can promote heparin-binding EGF release from the cell surface via activating Src family tyrosine kinases, and the activation of the EGF / EGFR signaling pathway eventually leads to epithelial-mesenchymal transition in AM.^44^ In addition to the epithelial cells of the uterine gland, a similar trend was observed in the myometrium. Myometrium has the ability of stem cell-like abnormal differentiation.^45^ Considering that the metaplasia of uterine smooth muscle cells was regarded as one crucial reason for the occurrence and development of AM,^46^ we speculate that GZFLW leads to the downregulation of the GPER / EGFR signaling pathway, which subsequently suppresses the estrogen resistance of smooth muscle cells and inhibits the abnormal differentiation of them.

In conclusion, based on the results of network pharmacology and animal experiments, we think that GZFLW can synergistically inhibit the abnormal proliferation and differentiation of AM via downregulating the ERα estrogen signaling pathway and suppress estrogen resistance of myocytes. This effect has no apparent interference on the normal endocrine.

# Conclusion

There are several limitations in our study. Firstly, the above databases we used can help predict the potential mechanism of GZFLW in treating AM. Nevertheless, most of them are based on the current research results, limiting the discovery of new therapeutic targets. Moreover, the primary detection indexes of the animal experiment only include ERα，GPER1 and EGFR, and it is not enough and comprehensive for data analysis. Finally, the number of animals is relatively small and may lead to study biases. Although network pharmacology strategy could help researchers simplify the complex system of TCM formulas and offer a novel research strategy for studying the mechanism of other TCM formulas, the mechanism remains confirmed by further experiments.

**Conflicts of interest**

The author(s) declare(s) that there is no conflict of interest regarding the publication of this paper.

**Funding Statement**

This study was supported by grants of National Natural Science Foundation of China (No.81973897), Scientific Research Foundation of HuBei provincial health department (No.WJ2019F079), and Yichang Medical and Health Research Foundation (No.A19-301-44).

**Supplementary Materials**

Supplementary Materials are provided. Table S1: detailed information of GZFLW compounds. Table S2: detailed information on therapeutic targets of AM. Table S3: detailed information of PPI network of 48 overlapped targets. Table S4: KEGG enrichment of overlapped targets from Metascape Database.

**References**

1.	Yu O, Schulze-Rath R, Grafton J, Hansen K, Scholes D, Reed SD. Adenomyosis incidence, prevalence and treatment: United States population-based study 2006-2015. *American journal of obstetrics and gynecology.* Jul 2020;223(1):94.e91-94.e10.

2.	Kossaï M, Penault-Llorca F. Role of Hormones in Common Benign Uterine Lesions: Endometrial Polyps, Leiomyomas, and Adenomyosis. *Advances in experimental medicine and biology.* 2020;1242:37-58.

3.	Zeng YY, Guan YG, Li KY. [Role of estrogen, estrogen receptors, and aromatase in the pathogenesis of uterine adenomyosis]. *Nan fang yi ke da xue xue bao = Journal of Southern Medical University.* Mar 20 2017;37(3):383-387.

4.	Baboo KD, Chen Z, Zhang X. [Progress on medical treatment in the management of adenomyosis]. *Zhejiang da xue xue bao. Yi xue ban = Journal of Zhejiang University. Medical sciences.* Apr 25 2019;48(2):142-147.

5.	Suardika A, Astawa Pemayun TG. New insights on the pathogenesis of endometriosis and novel non-surgical therapies. *Journal of the Turkish German Gynecological Association.* Aug 6 2018;19(3):158-164.

6.	Xiong Z, Lang L, Gao X, Xiao W, Wang Z, Zhao L. An integrative urinary metabolomic study of the therapeutic effect of Guizhi Fuling capsule on primary dysmenorrheal rats based (1)H NMR and UPLC-MS. *J Pharm Biomed Anal.* Feb 5 2019;164:750-758.

7.	Zheng W, Li M, Wang Y, et al. Guizhi Fuling Capsule Exhibits Antidysmenorrhea Activity by Inhibition of Cyclooxygenase Activity. 2020;2020:8607931.

8.	Hu S. *Effect of Guizhi Fuling pill combined with Marvelon on patients of qi stagnation and blood stasis type of Adenomyosis*, Hubei Univ Tradit Chin Med; 2015.

9.	Wang H, Zhou G, Zhuang M, Wang W, Fu X. Utilizing network pharmacology and molecular docking to explore the underlying mechanism of Guizhi Fuling Wan in treating endometriosis. *PeerJ.* 2021;9:e11087.

10.	Bahougne T, Angelopoulou E, Jeandidier N, Simonneaux V. Individual evaluation of luteinizing hormone in aged C57BL/6 J female mice. *GeroScience.* Feb 2020;42(1):323-331.

11.	He J, Hu GH. Progress on TCM Treatment for Dysmenorrhea due to Endometriosis ( Adenomyosis). *Chin Arch of Tradit Chin Med.* 2017;35(03):692-695.

12.	Du L, Du DH, Chen B, Ding Y, Zhang T, Xiao W. Anti-Inflammatory Activity of Sanjie Zhentong Capsule Assessed By Network Pharmacology Analysis of Adenomyosis Treatment. *Drug design, development and therapy.* 2020;14:697-713.

13.	Feng H, Zang L, Zhang DZ. Clinical Study of Modified Shaofu Zhuyu Decoction in Treating Adenomyosis Dysmenorrheal. *J Nanjing Univ Tradit Chin Med.* 2016;32(02):114-117.

14.	Wang YH. *Study on regulating the expression of ER α, ER β, GPER, PR-A and PR-B in mice with adenomyosis by clearing dampness and removing blood stasis*, Chengdu Univ Tradit Chin Med; 2015.

15.	Wang GQ, Gao Y, LIU F, Wei RL, Xie YM. Post-marketing surveillance on Guizhi Fuling Jiaonang based on literature review. *Chin J Chin Mater Med.* 2018;43(04):820-832.

16.	Galvankar M, Singh N, Modi D. Estrogen is essential but not sufficient to induce endometriosis. *Journal of biosciences.* Jun 2017;42(2):251-263.

17.	Chen P, Wang DB, Liang YM. Evaluation of estrogen in endometriosis patients: Regulation of GATA-3 in endometrial cells and effects on Th2 cytokines. *J Obstet Gynaecol Res.* Jun 2016;42(6):669-677.

18.	Piccinato CA, Neme RM, Torres N, et al. Effects of steroid hormone on estrogen sulfotransferase and on steroid sulfatase expression in endometriosis tissue and stromal cells. *J Steroid Biochem Mol Biol.* Apr 2016;158:117-126.

19.	Jin Z, Liu H, Xu C. Estrogen degrades Scribble in endometrial epithelial cells through E3 ubiquitin ligase HECW1 in the development of diffuse adenomyosis†. *Biology of reproduction.* Feb 14 2020;102(2):376-387.

20.	Hu R, Peng GQ, Ban DY, Zhang C, Zhang XQ, Li YP. High-Expression of Neuropilin 1 Correlates to Estrogen-Induced Epithelial-Mesenchymal Transition of Endometrial Cells in Adenomyosis. *Reproductive sciences (Thousand Oaks, Calif.).* Jan 2020;27(1):395-403.

21.	Chen Y, Wang J, Hong DY, et al. Baicalein has protective effects on the 17β-estradiol-induced transformation of breast epithelial cells. *Oncotarget.* Feb 7 2017;8(6):10470-10484.

22.	Nie J, Liu X. Quercetin alleviates generalized hyperalgesia in mice with induced adenomyosis. *Molecular medicine reports.* Oct 2017;16(4):5370-5376.

23.	Xu W, Song Y. Quercetin Inhibits Adenomyosis by Attenuating Cell Proliferation, Migration and Invasion of Ectopic Endometrial Stromal Cells. 2020;14:3815-3826.

24.	Ilhan M, Ali Z, Khan IA, Tastan H. The regression of endometriosis with glycosylated flavonoids isolated from Melilotus officinalis (L.) Pall. in an endometriosis rat model. *Taiwan J Obstet Gynecol.* 2020;59(2):211-219.

25.	Ilhan M, Ali Z, Khan IA, Tastan H, Akkol EK. Promising activity of Anthemis austriaca Jacq. on the endometriosis rat model and isolation of its active constituents. *Saudi Pharm J.* 2019;27(6):889-899.

26.	Park S, Lim W, Bazer FW, Whang KY, Song G. Quercetin inhibits proliferation of endometriosis regulating cyclin D1 and its target microRNAs in vitro and in vivo. *J Nutr Biochem.* Jan 2019;63:87-100.

27.	Schröder L, Marahrens P, Koch JG, et al. Effects of green tea, matcha tea and their components epigallocatechin gallate and quercetin on MCF‑7 and MDA-MB-231 breast carcinoma cells. *Oncology reports.* Jan 2019;41(1):387-396.

28.	Lee GA, Choi KC, Hwang KA. Kaempferol, a phytoestrogen, suppressed triclosan-induced epithelial-mesenchymal transition and metastatic-related behaviors of MCF-7 breast cancer cells. *Environmental toxicology and pharmacology.* Jan 2017;49:48-57.

29.	Xu Z, Zhao D, Zheng X, Huang B, Xia X, Pan X. Quercetin exerts bidirectional regulation effects on the efficacy of tamoxifen in estrogen receptor-positive breast cancer therapy: An in vitro study. Nov 2020;35(11):1179-1193.

30.	Guan YG, Liao JB, Li KY, Li YC. Potential mechanisms of an antiadenomyosis chinese herbal formula shaoyao-gancao decoction in primary cell culture model. 2014;2014:982913.

31.	Szubert M, Koziróg E, Olszak O, Krygier-Kurz K, Kazmierczak J, Wilczynski J. Adenomyosis and Infertility-Review of Medical and Surgical Approaches. Jan 30 2021;18(3).

32.	Singtripop T, Mori T, Sakamoto S, Sassa S, Park MK, Kawashima S. Suppression of the development of uterine adenomyosis by danazol treatment in mice. *Life sciences.* 1992;51(14):1119-1125.

33.	Nelson JF, Felicio LS, Randall PK, Sims C, Finch CE. A longitudinal study of estrous cyclicity in aging C57BL/6J mice: I. Cycle frequency, length and vaginal cytology. *Biology of reproduction.* Sep 1982;27(2):327-339.

34.	Wang YM, Wu MJ, Lin YH, Chen J. Association of epidermal growth factor receptor (EGFR) gene polymorphisms with endometriosis. *Medicine.* Apr 2019;98(17):e15137.

35.	Zhan H, Peng B, Ma J, et al. Epidermal growth factor promotes stromal cells migration and invasion via up-regulation of hyaluronate synthase 2 and hyaluronan in endometriosis. *Fertil Steril.* Oct 2020;114(4):888-898.

36.	Liu L, Luo N, Guo J, Xie Y, Chen L, Cheng Z. Berberine inhibits growth and inflammatory invasive phenotypes of ectopic stromal cells: Imply the possible treatment of adenomyosis. *Journal of pharmacological sciences.* May 2018;137(1):5-11.

37.	Wang T, Cao YG, Liu Y. Study on the mechanism of Guizhi Fuling Wan combined with Buzhong Yiqi Wan on benign prostatic hyperplasia in ovariectomized rats. *Chin ExpTradit Med Form.* 2010;16(17):154-157.

38.	Wang P, Henning SM, Magyar CE, Elshimali Y, Heber D, Vadgama JV. Green tea and quercetin sensitize PC-3 xenograft prostate tumors to docetaxel chemotherapy. *Journal of experimental & clinical cancer research : CR.* May 6 2016;35:73.

39.	Carazo Fernández A, Smutny T, Hyrsová L, Berka K, Pavek P. Chrysin, baicalein and galangin are indirect activators of the human constitutive androstane receptor (CAR). *Toxicology letters.* Mar 4 2015;233(2):68-77.

40.	Oi N, Chen H, Ok Kim M, Lubet RA, Bode AM, Dong Z. Taxifolin suppresses UV-induced skin carcinogenesis by targeting EGFR and PI3K. *Cancer prevention research (Philadelphia, Pa.).* Sep 2012;5(9):1103-1114.

41.	Khan KN, Fujishita A, Koshiba A, et al. Biological differences between focal and diffuse adenomyosis and response to hormonal treatment. *Reproductive biomedicine online.* Apr 2019;38(4):634-646.

42.	Hong DG, Park JY, Chong GO, et al. Transmembrane G protein-coupled receptor 30 gene polymorphisms and uterine adenomyosis in Korean women. *Gynecological endocrinology : the official journal of the International Society of Gynecological Endocrinology.* Jun 2019;35(6):498-501.

43.	Li JJ, Duan H. Expression Pattern of G-Protein-Coupled Estrogen Receptor in Myometrium of Uteri with and without Adenomyosis. 2017;2017:5974693.

44.	Chatterjee K, Jana S, DasMahapatra P, Swarnakar S. EGFR-mediated matrix metalloproteinase-7 up-regulation promotes epithelial-mesenchymal transition via ERK1-AP1 axis during ovarian endometriosis progression. *FASEB journal : official publication of the Federation of American Societies for Experimental Biology.* Aug 2018;32(8):4560-4572.

45.	Kishi Y, Shimada K, Fujii T, et al. Phenotypic characterization of adenomyosis occurring at the inner and outer myometrium. *PLoS One.* 2017;12(12):e0189522.

46.	García-Solares J, Donnez J, Donnez O, Dolmans MM. Pathogenesis of uterine adenomyosis: invagination or metaplasia? *Fertil Steril.* Mar 2018;109(3):371-379.
